# Copper acetate mediated thiomethylation of 2-pyridine-substituted acrylonitriles with DMSO

**DOI:** 10.1039/d5ra07270k

**Published:** 2025-10-16

**Authors:** Min Ye, Jie Yang, Cheng Huang, Zhengwang Chen

**Affiliations:** a Jiangxi Province Key Laboratory of Synthetic Pharmaceutical Chemistry, Gannan Normal University Ganzhou 341000 China yemin@gnnu.edu.cn chenzwang2021@163.com +86 797-8793670 +86 797-8793670

## Abstract

An efficient synthesis of a variety of alkenyl methyl thioethers from acrylonitriles and dimethyl sulfoxide is described. This copper acetate mediated thiomethylation reaction provides the corresponding products with broad substrate scope in moderate to excellent yields. This transformation is achieved through direct functionalization of vinylic C–H bonds, resulting in stereospecific formation of the formal cyanothiolation product of internal alkynes.

## Abstract 2

An efficient synthesis of alkenyl methyl thioethers through direct C–H functionalization from acrylonitriles with DMSO as the methylthio source.

## Graphical abstract

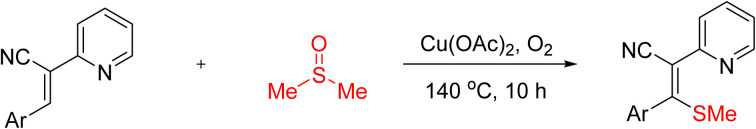

Organic molecules containing C–S bonds are widely distributed in nature and exhibit diverse valuable biological activities.^1^ Consequently, significant efforts have been devoted to developing efficient synthetic methodologies for C–S bond formation.^2^ Among these protocols, transition-metal-catalyzed coupling reactions between vinyl/aryl halides and thiols, sulfonyl chlorides, or disulfides have emerged as prominent strategies in recent decades.^3^ However, these methods predominantly suffer from the requirement of pre-functionalized substrates. Over the past decades, metal-catalyzed, directing-group mediated selective C–H functionalization has emerged as a powerful strategy in an energy-efficient and step-economic fashion.^4^ The majority of advancements in C(sp^2^)−H functionalization research have predominantly centered on arenes.^5^ In contrast, synthetically viable methodologies for metal-catalyzed direct C–H functionalization of olefins remain relatively underdeveloped.^6^ Direct activation of non-aromatic vinylic C–H bonds presents significant challenges, because of the increased reactivity and lability of olefinic systems. For these reasons, direct C–S bond formation *via* C–H functionalization from olefin derivatives have emerged as an attractive and challenging goal.

Dimethyl sulfoxide (DMSO) is an inexpensive organic sulfur compound, characterized as a colorless, odorless, and tasteless high-boiling-point liquid. This polar aprotic solvent has been widely utilized in organic synthesis, such as in Swern oxidation, Pfitzner-Moffatt oxidation, and Corey–Chaykovsky reaction.^7^ Moreover, DMSO has been reported as important sources for O,^8^ Me,^9^ SMe,^10^ SOCH_3_,^11^ SO_2_Me,^12^ CN^13^ and CHO^14^ groups in organic reactions. Among the transformations, Jain group reported a copper acetate-DMSO promoted methylthiolation of arenes and heteroarenes.^15^ Compared to using DMSO to form aryl methyl thioethers through C–H functionalization,^16^ the direct formation of alkenyl methyl thioethers is considerably less studied. Therefore, the development of new transformation toward alkenyl methyl thioethers by using easily available substrates and cheap DMSO with high efficiency would be highly desiable.

Vinyl nitriles represent the predominant structural scaffold found in diverse chemical systems, including pharmaceuticals, dyes, agrochemicals, herbicides, and natural product.^17^ Beyond their established roles in biological systems, these compounds serve as highly adaptable synthetic intermediates in organic chemistry, which can be readily converted into various important functional groups such as acrylic acid derivatives, aldehydes, amines, nitrogen-based heterocycles, *etc.*^18^ Direct functionalization of the alkene double bond in acrylonitriles *via* C–H bond activation enables the formation of valuable difunctional compounds. This transformation represents a powerful strategy for constructing complex molecules through selective modifications of the α,β-unsaturated nitrile system. Very recently, we have described highly efficient approaches for the synthesis of nitrogen-containing fused heterocycles^19^ and diarylfumaronitriles^20^ from alkenylnitriles. As part of this continuing project of the functionalization of aryl-substituted acrylonitriles, here we present a copper-mediated thiomethylation of 2-pyridine-substituted acrylonitriles with DMSO (Scheme 1). Although the cyanothiolation of internal alkynes represents a more direct synthetic approach, controlling stereoselectivity remains challenging.^21^

**Scheme 1:**
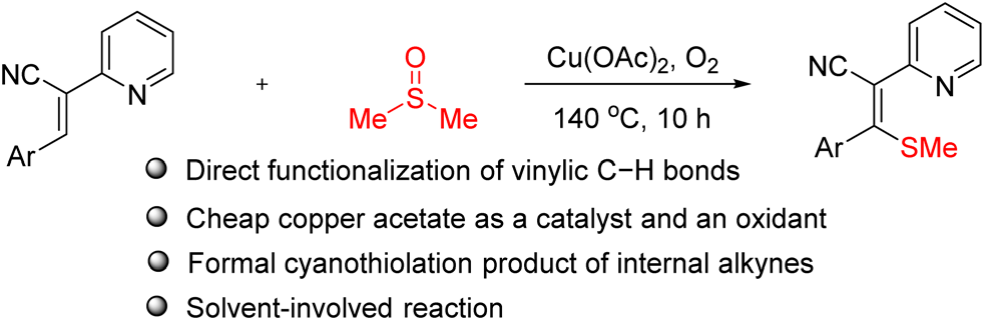
The direct thiomethylation of acrylonitriles with DMSO.

Initially, diarylacetonitrile 1a was employed as the substrate for condition optimization (Table 1). DMSO served as both the methylthiolation reagent and solvent. Based on literature reports indicating iodide-promoted C–H bond methylthiolation with DMSO,^22^ various iodide-containing reagents including *N*-iodosuccinimide, iodine, potassium iodide, and ammonium iodide were tested, but the desired product 3a was not obtained (entries 1–4). Copper oxide and copper salt were also screened without success (entries 5–6). Notably, when copper acetate was used, the target product 3a was formed, albeit with a modest yield of 54% (entry 7), suggesting the catalytic potential of Cu(OAc)_2_. Given the significant impact of catalyst and oxidant loading, the amount of Cu(OAc)_2_ was systematically investigated. Reducing the stoichiometry to 0.3 equiv resulted in complete suppression of the reaction (entry 8), while 0.5 equiv afforded 3a in 37% yield (entry 9). Further optimization revealed that 2 equiv of Cu(OAc)_2_ led to the highest yield (entries 10–12). Considering oxygen is an ideal green oxidant, reaction was conducted under O_2_ atmospheres. Remarkably, O_2_ atmosphere boosted the yield to 92% (entry 13). Temperature optimization demonstrated that deviations from 140 °C (either 130 °C or 150 °C) reduced yields to 74% and 71%, respectively (entries 14–15).

**Table 1:** Optimization of the reaction conditions^a^

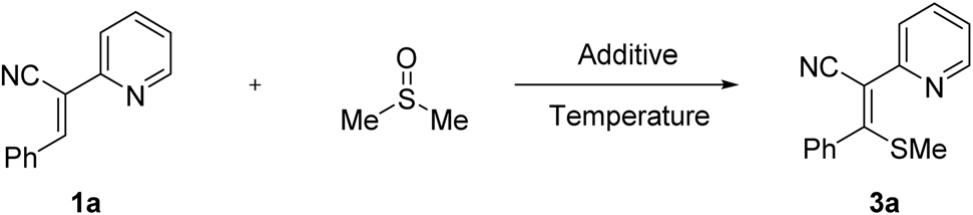			
Entry	Additive (X eq.)	Temperature (^o^C)	Yield^b^ (%)
1	NIS (2)	140	n.p
2	I_2_ (2)	140	n.p
3	KI (2)	140	n.p
4	NH_4_I (2)	140	n.p
5	CuO (0.8)	140	n.p
6	CuI (0.8)	140	n.p
7	Cu(OAc)_2_ (0.8)	140	54
8	Cu(OAc)_2_ (0.3)	140	Trace
9	Cu(OAc)_2_ (0.5)	140	37
10	Cu(OAc)_2_ (1)	140	68
11	Cu(OAc)_2_ (2)	140	88
12	Cu(OAc)_2_ (3)	140	85
13^c^	Cu(OAc)_2_ (2)	140	92
14	Cu(OAc)_2_ (2)	130	74
15	Cu(OAc)_2_ (2)	150	71

a Reaction conditions: 1a (0.1 mmol) and additive (0.5–3 equiv) with DMSO (1.5 mL) for 10 h in air atmosphere.

b Isolated yield.

c Under O_2_.

Under the optimized conditions, the substrate scope was systematically explored (Scheme 2). The reaction demonstrated excellent functional group tolerance. Substrates bearing various electron-donating (EDGs) and electron-withdrawing groups (EWGs) on the aromatic ring smoothly afforded the corresponding products (**3b–3l**). Notably, *ortho*-methyl-substituted substrate also obtained high yield compared to the *para*-counterpart, suggesting negligible steric hindrance in this transformation (**3b–3c**). Similarly, bulky *tert*-butyl substituents were well tolerated, furnishing the desired products in satisfactory yield (3d). Strong EDG, such as methoxy group, significantly promoted the reaction. Remarkably, even tri-methoxylated substrate proceeded smoothly, highlighting the beneficial effect of EDGs (**3f–3g**). Regarding EWGs, moderate yields were observed for substrates containing halogens (I/Br) at the *ortho* position (**3i–3j**). Importantly, these halogenated products could serve as versatile platforms for subsequent transition-metal-catalyzed cross-coupling reactions, enabling further functionalization. Substrates with trifluoromethyl groups, which are pharmacologically relevant, also underwent the reaction efficiently (3l). Beyond simple aromatic rings, extended π-conjugated systems such as naphthalene and anthracene derivatives were successfully compatible (**3m–3o**). Pyridine substrates with methyl substituents also participated in the reaction (3p). Noteworthily, the reaction could be carried out at a 2 mmol scale and afforded the product with satisfactory yield (3a). Unluckily, aliphatic substituted substrate failed to afford the corresponding product. These results implied that the thiomethylation reaction can be effective for the alkenyl methyl thioether library.

**Scheme 2:**
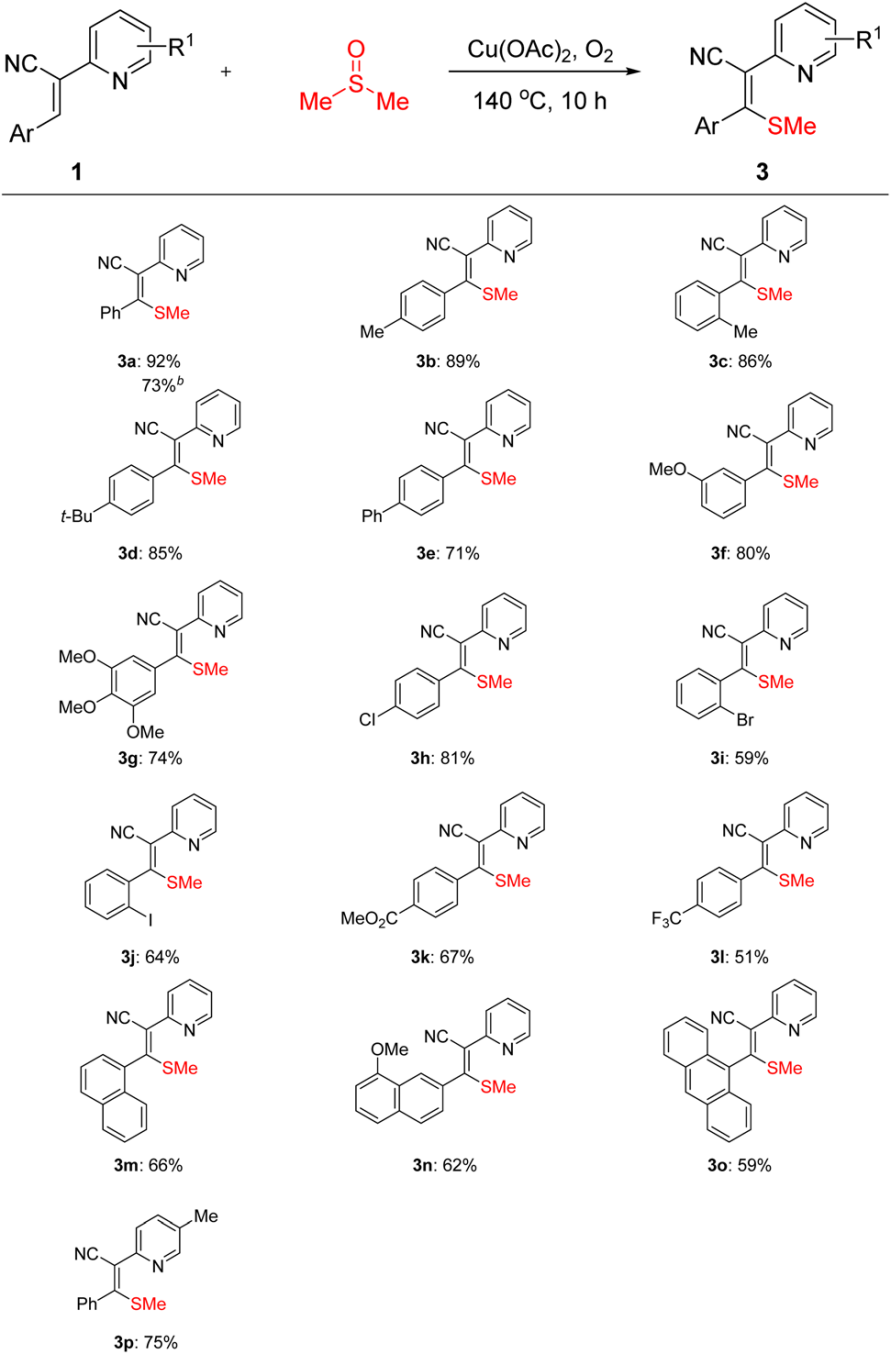
Substrate scope of 2-pyridine-substituted acrylonitriles^*a*^. ^*a*^Reaction conditions: 1 (0.1 mmol) and Cu(OAc)_2_ (2 equiv) with DMSO (1.5 mL) at 140 °C for 10 h in an oxygen atmosphere; isolated yield. ^*b*^2 mmol scale of the reaction.

To elucidate the reaction mechanism, a series of control experiments were conducted in Scheme 3. Initially, 2,2,6,6-tetramethyl-1-piperidinyloxy (TEMPO) was added under standard conditions for radical-trapping experiment, and it was found that it had little effect on the yield of 3a (Scheme 3a). It means that radical intermediate may not be generated in the reaction. Subsequently, substrates 1q and 1r, featuring nitrogen atoms at different positions of the pyridine ring, failed to yield the corresponding methylthiolation products (Scheme 3b and c). This suggests that the pyridyl nitrogen at the 2-position coordinates with the metal center, forming a metallacyclic intermediate that activates the C–H bond. When the cyano group was changed into ester group, substrate 1s also failed to react, demonstrating the crucial role of the cyano group in facilitating the transformation (Scheme 3d). The deuterium-labeling experiment with DMSO-*d*_6_ demonstrated that the methylthio group originates from DMSO (Scheme 3e). When *n*-butanthiol or diethyldisulfide was introduced to the reaction mixture under standard conditions, both the target product 3a and the corresponding 3u or 3v were obtained (Scheme 3f and g). Based on these findings, it can be inferred that dimethyl sulfoxide is likely converted into methanthiol and dimethyldisulfide during the reaction process, and dialkyl disulfide was the actual thioalkylating agent.

**Scheme 3:**
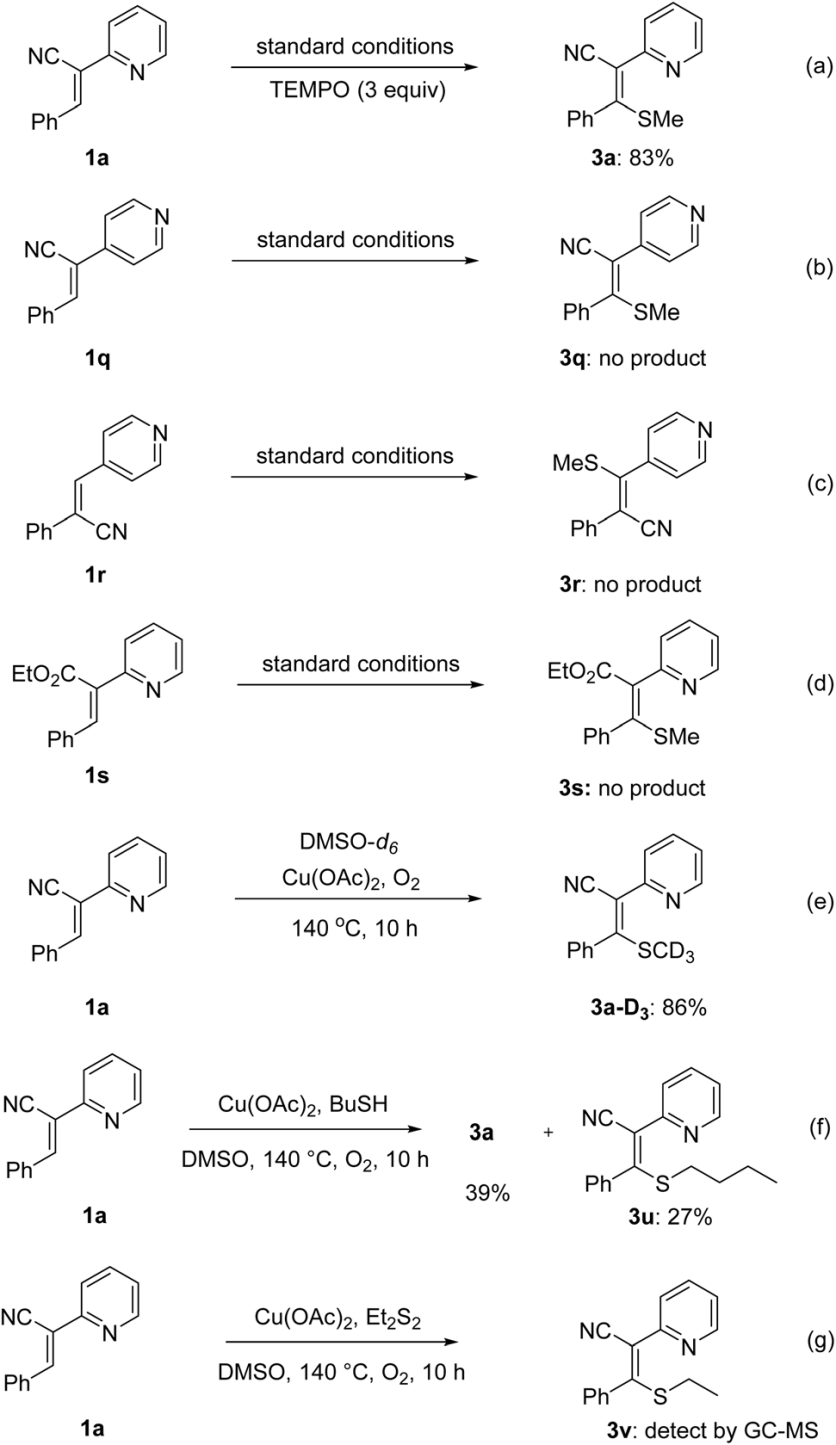
Control experiments.

Based on previous literature reports and our experimental results,^15,16^ a plausible reaction mechanism is proposed (Scheme 4). Initially, dimethyl sulfoxide undergoes thermal decomposition to generate methanthiol. Subsequently, anion exchange with copper acetate produces intermediate A. The active catalyst A then coordinates with substrate 1a and activates the olefinic C–H bond to form intermediate B, which eliminates one molecule of acetic acid to yield intermediate C. Cu(OAc)_2_ oxidizes Cu(ii) to generate the Cu(iii) intermediate D. Finally, reductive elimination affords product 3a and Cu(i), which is reoxidized by molecular oxygen to regenerate Cu(ii) and complete the catalytic cycle. In this process, copper acetate serves dual roles as both catalyst and oxidant.

**Scheme 4:**
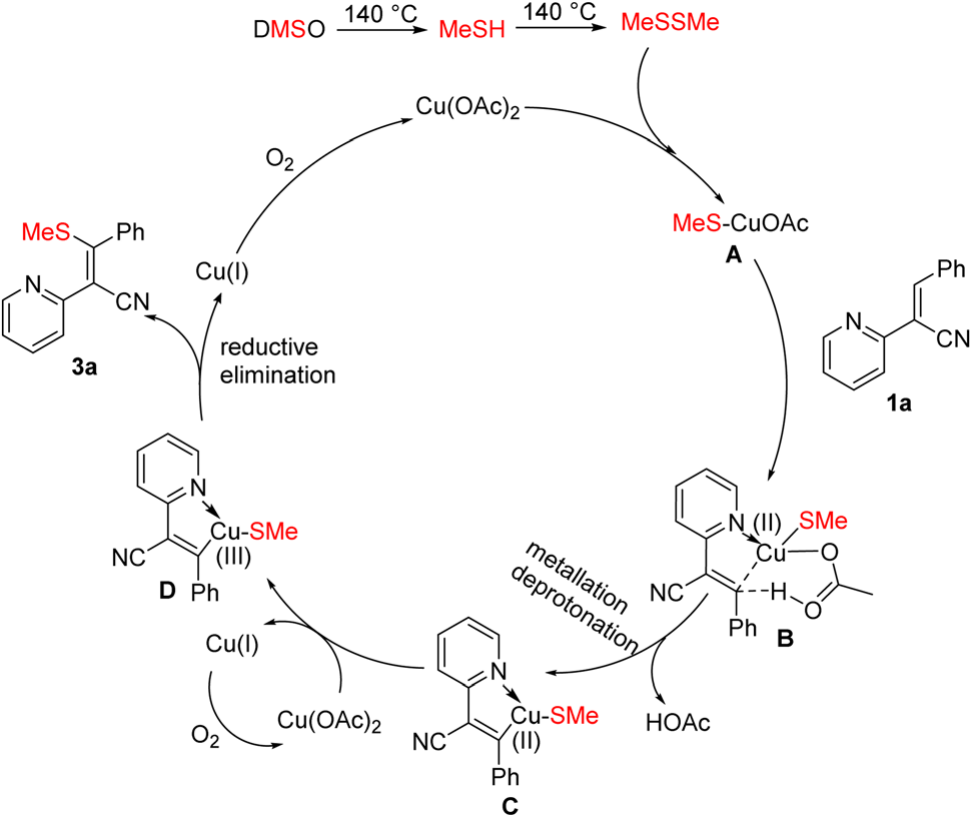
Possible reaction mechanism.

In summary, we have developed a novel methodology for the direct functionalization of C–H bonds in 2-pyridyl acrylonitriles, enabling the construction of C–S bonds. Stoichiometric copper acetate serves a dual role as both a catalyst for C–H bond functionalization and oxidant with molecular oxygen. The inexpensive organic solvent dimethyl sulfoxide (DMSO) functions as the methylthio group source. Studies regarding the mechanism and application are currently ongoing in our laboratory.

## Conflicts of interest

There are no conflicts to declare.

## Supplementary Material

RA-015-D5RA07270K-s001

## Data Availability

The data supporting this article have been included as part of the supplementary information (SI). Supplementary information: experimental section, characterization of all compounds, copies of ^1^H and ^13^C NMR spectra for selected compounds. See DOI: https://doi.org/10.1039/d5ra07270k.
