# Supplementary material for: Copper acetate mediated thiomethylation of 2-pyridine-substituted acrylonitriles with DMSO
Source: RSC Adv. 2025 Oct 16;15(46):38832–5. doi: 10.1039/d5ra07270k (PMC12529248; doi:10.1039/d5ra07270k)
Supplement: RA-015-D5RA07270K-s001 [file RA-015-D5RA07270K-s001.pdf]

Supplementary Information (SI) for RSC Advances.
This journal is © The Royal Society of Chemistry 2025

Copper acetate mediated thiomethylation of 2-pyridine-substituted acrylonitriles with DMSO

Min Ye,* Jie Yang, Cheng Huang, Zhengwang Chen*

Jiangxi Province Key Laboratory of Synthetic Pharmaceutical Chemistry, Gannan Normal University, Ganzhou 341000, PR China.

E-mail: yemin@gnnu.edu.cn; chenzwang2021 @163.com

Supporting Information

List of Contents

A. General method 2S

B. Preparation of starting materials 2S

C. General procedure for the thiomethylation of 2-pyridine-substituted acrylonitriles with DMSO 2S

D. Analytical data 3S

E. Reference 8S

F. Copies of ^{1}H and ^{13}C NMR spectra 9S

A. General method

Melting points were investigated using a melting point instrument and are uncorrected. ^{1}H and ^{13}C NMR spectra were obtained on a 400 MHz for ^{1}H NMR and 100 MHz for ^{13}C NMR. The chemical shifts are referenced to signals at 7.26 and 77.0 ppm, respectively, chloroform is solvent with TMS as the internal standard unless otherwise noted. High resolution mass spectra (HRMS) (TOF) were measured using an electrospray ionization (ESI) mass spectrometry. Silica gel (300-400 mesh) was used for flash column chromatograph, eluting (unless otherwise stated) with ethyl acetate/petroleum ether (PE) (60-90 ºC) mixture.

B. Preparation of starting materials

The route toward 2-pyridine-substituted acrylonitriles:

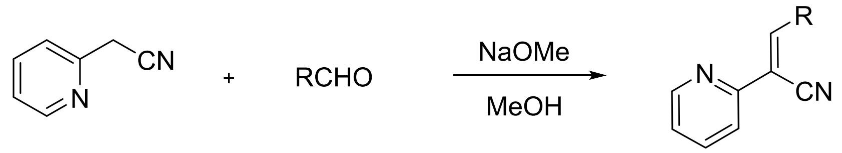

A' **1**

Method: Following a known procedure,[1] 2-pyridine-substituted acrylonitriles were synthesized.

To a solution of 2-pyridylacetonitrile (118 mg, 1 mmol) and aldehyde **A’** (1 mmol) in MeOH (5 mL) was added NaOMe (5.4 mg, 0.1 mmol), and the reaction mixture was stirred at room temperature for 3 h. After the reaction was finished, water (5 mL) was added and the solution was extracted with ethyl acetate (3×10 mL), and the combined extract was dried with anhydrous $MgSO_4$. Solvent was removed, and the residue was purified by column chromatography to give the pure acrylonitriles **1**.

C. General procedure for the thiomethylation of 2-pyridine-substituted acrylonitriles with DMSO

A mixture of 2-pyridine-substituted acrylonitrile (0.1 mmol) and $Cu(OAc)_2$ (36 mg, 2 equiv) in DMSO (1.5 mL) was stirred in a preheated oil bath at 140 °C for 10 h in a sealed tube under 1 atm of oxygen. After the reaction was finished, water (5 mL) was added, the solution was extracted with ethyl acetate (3 × 5 mL), and the combined extract was dried with anhydrous $MgSO_4$. The solvent was removed, and the residue was separated by column chromatography to give the pure sample.

D. Analytical data

(*E*)-3-(methylthio)-3-phenyl-2-(pyridin-2-yl)acrylonitrile (3a)

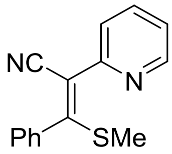

Brown soild; mp = 119-120 °C; R_f = 0.40 (petroleum ether / ethyl acetate = 7:1); ^{1}H NMR (400 MHz, $CDCl_3$) δ = ^{1}H NMR (400 MHz, $CDCl_3$) δ 8.72 (d, J = 4.8 Hz, 1H), 7.82 – 7.72 (m, 2H), 7.54 – 7.44 (m, 3H), 7.37 (dd, J = 5.2, 3.2 Hz, 2H), 7.26 – 7.22 (m, 1H), 1.86 (s, 3H). ^{13}C NMR (100 MHz, $CDCl_3$) δ = 164.91, 152.52, 148.04, 136.84, 136.70, 129.41, 129.02, 128.16, 122.97, 122.09, 118.12, 107.33, 17.52. HRMS (ESI): calcd. for $C_{15}H_{13}N_2S$ $[M + H]^+$ 253.0794, found 253.0794.

(*E*)-3-(methylthio)-2-(pyridin-2-yl)-3-(*p*-tolyl)acrylonitrile (3b)

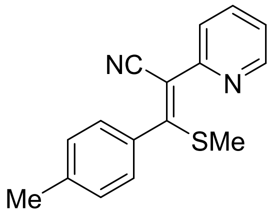

Brown soild; mp = 128-130 °C; R_f = 0.40 (petroleum ether / ethyl acetate = 7:1); ^{1}H NMR (400 MHz, $CDCl_3$) δ = 8.71 (d, J = 4.7 Hz, 1H), 7.75 (qd, J = 6.6, 1.3 Hz, 2H), 7.30 (d, J = 8.0 Hz, 2H), 7.25 (s, 1H), 7.25 – 7.20 (m, 2H), 2.41 (s, 3H), 1.86 (s, 3H). ^{13}C NMR (100 MHz, $CDCl_3$) δ = 165.24, 152.60, 148.04, 139.48, 136.65, 133.88, 129.69, 128.09, 122.98, 122.02, 118.32, 107.23, 21.38, 17.54. HRMS (ESI): calcd. for $C_{16}H_{15}N_2S$ $[M + H]^+$ 267.0950, found 267.0953.

(*E*)-3-(methylthio)-2-(pyridin-2-yl)-3-(*o*-tolyl)acrylonitrile (3c)

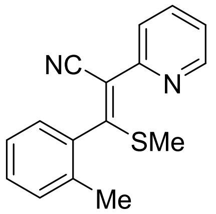

Yellow liquid; R_f = 0.47 (petroleum ether / ethyl acetate = 7:1); ^{1}H NMR (400 MHz, $CDCl_3$) δ = 8.75 – 8.71 (m, 1H), 7.75 (td, J = 6.7, 1.3 Hz, 2H), 7.36 – 7.31 (m, 3H), 7.24 – 7.17 (m, 2H), 2.36 (s, 3H), 1.78 (s, 3H). ^{13}C NMR (100 MHz, $CDCl_3$) δ = 164.99, 152.49, 147.90, 136.67, 136.51, 134.79, 130.69, 129.39, 127.87, 126.56, 122.51, 121.80, 117.73, 106.47, 18.95, 16.82. HRMS (ESI): calcd. for $C_{16}H_{15}N_2S$ $[M + H]^+$ 267.0950, found 267.0953.

(*E*)-3-(4-(tert-butyl)phenyl)-3-(methylthio)-2-(pyridin-2-yl)acrylonitrile (3d)

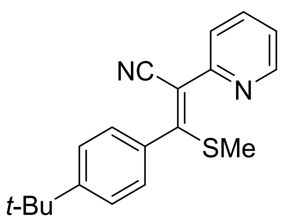

Brown soild; mp = 88-90 °C; R_f = 0.48 (petroleum ether / ethyl acetate = 7:1); ^{1}H NMR (400 MHz, $CDCl_3$) δ = 8.73 – 8.70 (m, 1H), 7.78 – 7.73 (m, 2H), 7.52 – 7.49 (m, 2H), 7.32 – 7.28 (m, 2H), 7.23 (ddd, J = 6.7, 4.9, 1.9 Hz, 1H), 1.85 (s, 3H), 1.36 (s, 9H). ^{13}C NMR (100 MHz, $CDCl_3$) δ = 165.19, 152.68, 152.58, 148.07, 136.64, 133.74, 127.98, 125.83, 123.07, 122.05, 118.29, 107.20, 34.79, 31.23, 17.53. HRMS (ESI): calcd. for $C_{19}H_{21}N_2S$ [M + H]$^+$ 309.1420, found 309.1421.

(*E*)-3-(4-(*tert*-butyl)phenyl)-3-(methylthio)-2-(pyridin-2-yl)acrylonitrile (3e)

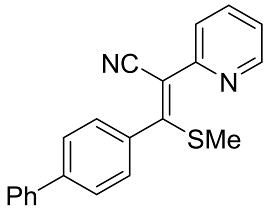

Brown soild; mp = 107-109 °C; R_f = 0.30 (petroleum ether / ethyl acetate = 7:1); ^{1}H NMR (400 MHz, $CDCl_3$) δ = 8.76 – 8.72 (m, 1H), 7.80 – 7.78 (m, 2H), 7.75 – 7.73 (m, 2H), 7.65 (d, J = 7.0 Hz, 2H), 7.46 (dd, J = 8.3, 2.3 Hz, 4H), 7.40 (d, J = 7.3 Hz, 1H), 7.26 – 7.23 (m, 1H), 1.92 (s, 3H). ^{13}C NMR (100 MHz, $CDCl_3$) δ = 164.61, 152.49, 148.05, 136.67, 135.57, 130.47, 128.82, 128.71, 127.78, 127.58, 127.48, 127.08, 123.00, 122.11, 118.19, 107.37, 17.62. HRMS (ESI): calcd. for $C_{21}H_{17}N_2S$ [M + H]$^+$ 329.1107, found 329.1107.

(*E*)-3-(3-methoxyphenyl)-3-(methylthio)-2-(pyridin-2-yl)acrylonitrile (3f)

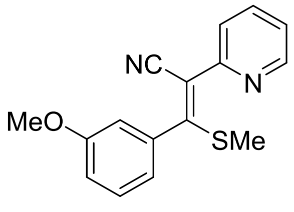

Brown soild; mp = 104-106 °C; R_f = 0.30 (petroleum ether / ethyl acetate = 7:1); ^{1}H NMR (400 MHz, $CDCl_3$) δ = 8.71 (d, J = 4.7 Hz, 1H), 7.79 – 7.72 (m, 2H), 7.42 (t, J = 7.9 Hz, 1H), 7.24 (ddd, J = 6.7, 4.9, 1.5 Hz, 1H), 6.99 – 6.93 (m, 2H), 6.90 – 6.87 (m, 1H), 3.85 (s, 3H), 1.90 (s, 3H). ^{13}C NMR (100 MHz, $CDCl_3$) δ = 164.63, 159.91, 152.48, 148.03, 137.94, 136.71, 130.17, 122.96, 122.10, 120.42, 118.04, 115.03, 113.63, 107.19, 55.36, 17.37. HRMS (ESI): calcd. for $C_{16}H_{15}N_2OS$ [M + H]$^+$ 283.0900, found 283.0900.

(*E*)-3-(methylthio)-2-(pyridin-2-yl)-3-(3,4,5-trimethoxyphenyl)acrylonitrile (3g)

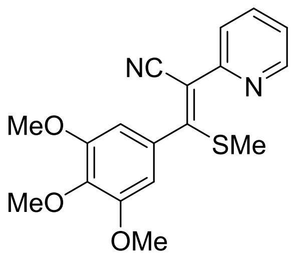

Brown soild; mp = 89-90 °C; R_f = 0.34 (petroleum ether / ethyl acetate = 3:1); ^{1}H NMR (400 MHz, $CDCl_3$) δ = 8.72 (d, J = 4.8 Hz, 1H), 7.82 – 7.72 (m, 2H), 7.54 – 7.44 (m, 3H), 7.37 (dd, J = 5.2, 3.2 Hz, 2H), 7.26 – 7.22 (m, 1H), 1.86 (s, 3H). ^{13}C NMR (100 MHz, $CDCl_3$) δ = 64.57, 153.67, 152.49, 148.18, 138.81, 136.71, 131.89, 123.13, 122.24, 118.13, 107.48, 105.64, 61.01, 56.33, 17.37. HRMS (ESI): calcd. for $C_{18}H_{19}N_2O_3S$ [M + H]$^+$ 343.1111, found 343.1111.

(*E*)-3-(4-chlorophenyl)-3-(methylthio)-2-(pyridin-2-yl)acrylonitrile (3h)

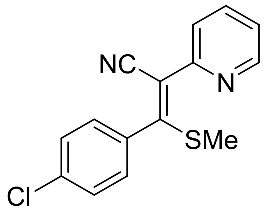

Brown liquid; R_f = 0.29 (petroleum ether / ethyl acetate = 7:1); ^{1}H NMR (400 MHz, $CDCl_3$) δ = 8.72 (d, J = 4.3 Hz, 1H), 7.80 – 7.72 (m, 2H), 7.52 – 7.48 (m, 2H), 7.34 – 7.30 (m, 2H), 7.26 (dd, J = 4.3, 1.8 Hz, 1H), 1.87 (s, 3H). ^{13}C NMR (100 MHz, $CDCl_3$) δ = 163.37, 152.25, 148.08, 136.77, 135.63, 135.19, 129.65, 129.43, 123.03, 122.28, 117.94, 107.78, 17.64. HRMS (ESI): calcd. for $C_{15}H_{12}ClN_2S$ [M + H]$^+$ 287.0404, found 287.0402.

(*E*)-3-(2-bromophenyl)-3-(methylthio)-2-(pyridin-2-yl)acrylonitrile (3i)

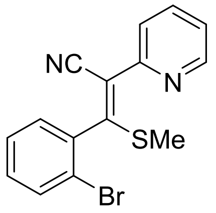

Brown liquid; R_f = 0.33 (petroleum ether / ethyl acetate = 7:1); ^{1}H NMR (400 MHz, $CDCl_3$) δ = 8.74 (d, J = 4.8 Hz, 1H), 7.78 (dt, J = 6.3, 3.3 Hz, 2H), 7.74 – 7.68 (m, 1H), 7.50 – 7.46 (m, 1H), 7.37 – 7.30 (m, 2H), 7.26 – 7.22 (m, 1H), 1.88 (s, 3H). ^{13}C NMR (100 MHz, $CDCl_3$) δ = 163.29, 152.28, 148.02, 137.96, 136.76, 133.37, 130.79, 129.65, 128.22, 128.17, 122.75, 122.07, 117.44, 107.48, 16.97. HRMS (ESI): calcd. for $C_{15}H_{12}BrN_2S$ [M + H]$^+$ 330.9899, found 330.9898.

(*E*)-3-(2-iodophenyl)-3-(methylthio)-2-(pyridin-2-yl)acrylonitrile (3j)

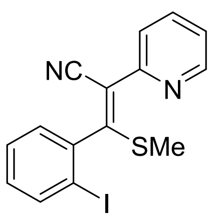

Brown soild; mp = 129-131 °C; R_f = 0.37 (petroleum ether / ethyl acetate = 7:1); ^{1}H NMR (400 MHz, $CDCl_3$) δ = 8.74 (dd, J = 3.5, 1.3 Hz, 1H), 7.98 (dd, J = 8.0, 0.9 Hz, 1H), 7.80 – 7.75 (m, 2H), 7.52 (td, J = 7.6, 1.1 Hz, 1H), 7.33 (dd, J = 7.6, 1.6 Hz, 1H), 7.26 – 7.23 (m, 1H), 7.15 (td, J = 7.8, 1.6 Hz, 1H), 1.87 (s, 3H). ^{13}C NMR (100 MHz, $CDCl_3$) δ = 165.93, 152.31, 148.07, 141.97, 139.80, 136.77, 130.58, 129.03, 128.79, 122.76, 122.08, 117.41, 107.55, 97.41, 17.15. HRMS (ESI): calcd. for $C_{15}H_{12}IN_2S$ [M + H]$^+$ 378.9760, found 378.9760.

methyl (E)-4-(2-cyano-1-(methylthio)-2-(pyridin-2-yl)vinyl)benzoate (3k)

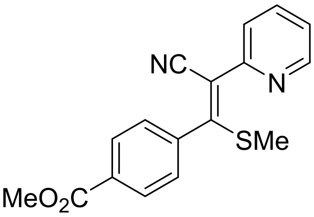

Brown soild; mp = 139-140 °C; R_f = 0.23 (petroleum ether / ethyl acetate = 7:1); ^{1}H NMR (400 MHz, $CDCl_3$) δ = 8.73 (d, J = 4.7 Hz, 1H), 8.19 (d, J = 8.3 Hz, 2H), 7.81 – 7.74 (m, 2H), 7.46 (d, J = 8.3 Hz, 2H), 7.28 (d, J = 1.2 Hz, 1H), 3.96 (s, 3H), 1.85 (s, 3H). ^{13}C NMR (100 MHz, $CDCl_3$) δ = 166.27, 163.44, 152.25, 148.08, 141.25, 136.81, 131.06, 130.39, 128.40, 123.01, 122.32, 117.77, 107.61, 52.35, 17.58. HRMS (ESI): calcd. for $C_{17}H_{15}N_2O_2S$ [M + H]$^+$ 311.0849, found 311.0849.

(*E*)-3-(methylthio)-2-(pyridin-2-yl)-3-(4-(trifluoromethyl)phenyl)acrylonitrile (3l)

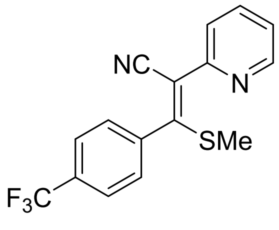

Yellow solid; mp = 121-122 °C; R_f = 0.25 (petroleum ether / ethyl acetate = 7:1); ^{1}H NMR (400 MHz, $CDCl_3$) δ = 8.73 (d, J = 4.5 Hz, 1H), 7.81 – 7.74 (m, 4H), 7.52 (d, J = 8.0 Hz, 2H), 7.29 – 7.26 (m, 1H), 1.85 (s, 3H). ^{13}C NMR (100 MHz, $CDCl_3$) δ = 162.77, 152.13, 148.10, 140.38, 136.84, 131.66 (q, J = 4 Hz), 128.82, 126.19 (q, J = 4 Hz), 123.68 (q, J = 271 Hz), 123.07, 122.43, 117.69, 107.95, 17.65. HRMS (ESI): calcd. for $C_{16}H_{12}F_3N_2S$ [M + H]$^+$ 321.0668, found 321.0668.

(*E*)-3-(methylthio)-3-(naphthalen-1-yl)-2-(pyridin-2-yl)acrylonitrile (3m)

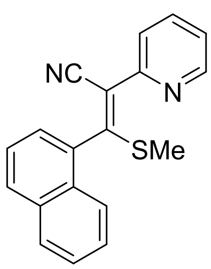

Brown soild; mp = 121-123 °C; R_f = 0.33 (petroleum ether / ethyl acetate = 4:1); ^{1}H NMR (400 MHz, $CDCl_3$) δ = 8.79 (dt, J = 4.8, 1.3 Hz, 1H), 8.01 – 7.91 (m, 3H), 7.80 (dd, J = 4.9, 1.4 Hz, 2H), 7.60 (dd, J = 8.2, 7.1 Hz, 1H), 7.54 (ddd, J = 6.8, 3.9, 1.9 Hz, 2H), 7.46 (dd, J = 7.0, 1.1 Hz, 1H), 7.29 – 7.26 (m, 1H), 1.69 (s, 3H). ^{13}C NMR (100 MHz, $CDCl_3$) δ = 163.83, 152.63, 147.97, 136.76, 134.38, 133.53, 130.33, 129.83, 128.63, 127.45, 126.66, 126.24, 125.42, 124.15, 122.72, 121.97, 117.84, 107.76, 16.75. HRMS (ESI): calcd. for $C_{19}H_{15}N_2S$ $[M + H]^+$ 303.0950, found 303.0950.

(*E*)-3-(8-methoxynaphthalen-2-yl)-3-(methylthio)-2-(pyridin-2-yl)acrylonitrile (3n)

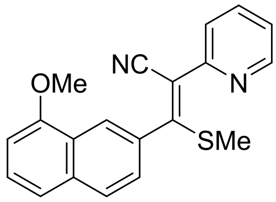

Brown soild; mp = 136-137 °C; R_f = 0.23 (petroleum ether / ethyl acetate = 7:1); ^{1}H NMR (400 MHz, $CDCl_3$) δ = 8.75 (d, J = 4.7 Hz, 1H), 7.87 (d, J = 8.5 Hz, 1H), 7.84 – 7.76 (m, 4H), 7.43 (dd, J = 8.4, 1.8 Hz, 1H), 7.26 – 7.17 (m, 3H), 3.95 (s, 3H), 1.87 (s, 3H). ^{13}C NMR (100 MHz, $CDCl_3$) δ = 165.06, 158.76, 152.70, 148.12, 136.70, 134.81, 131.90, 130.02, 128.50, 127.73, 127.70, 126.08, 123.09, 122.12, 119.76, 118.35, 107.62, 105.83, 55.42, 17.60. HRMS (ESI): calcd. for $C_{20}H_{17}N_2OS$ $[M + H]^+$ 333.1056, found 333.1056.

(E)-3-(anthracen-9-yl)-3-(methylthio)-2-(pyridin-2-yl)acrylonitrile (3o)

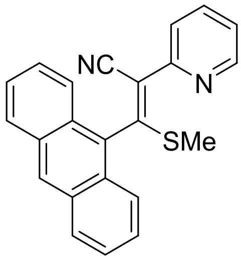

Yellow solid; mp = 198-200 °C; R_f = 0.40 (petroleum ether / ethyl acetate = 7:1); ^{1}H NMR (400 MHz, $CDCl_3$) δ = 8.86 (dt, J = 4.9, 1.4 Hz, 1H), 8.58 (s, 1H), 8.08 (dd, J = 7.6, 2.2 Hz, 4H), 7.83 – 7.80 (m, 2H), 7.56 – 7.50 (m, 4H), 7.30 (dd, J = 8.9, 4.5 Hz, 1H), 1.45 (s, 3H). ^{13}C NMR (100 MHz, $CDCl_3$) δ = 162.81, 152.78, 148.03, 136.81, 131.17, 129.87, 129.04, 128.92, 128.62, 127.43, 125.69, 124.31, 122.63, 121.98,

117.58, 108.55, 16.22. HRMS (ESI): calcd. for $C_{23}H_{17}N_2S$ $[M + H]^+$ 353.1107, found 353.1107.

(*E*)-2-(5-methylpyridin-2-yl)-3-(methylthio)-3-phenylacrylonitrile (3p)

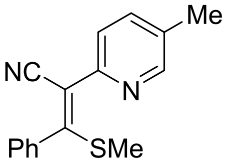

Brown soild; mp = 132-134 °C; R_f = 0.43 (petroleum ether / ethyl acetate = 7:1); ^1H NMR (400 MHz, $CDCl_3$) δ = 8.55 (s, 1H), 7.64 (d, J = 8.0 Hz, 1H), 7.58 (dd, J = 8.2, 2.1 Hz, 1H), 7.50 (ddd, J = 7.4, 6.2, 1.4 Hz, 2H), 7.47 – 7.44 (m, 1H), 7.39 – 7.35 (m, 2H), 2.38 (s, 3H), 1.85 (s, 3H). ^{13}C NMR (100 MHz, $CDCl_3$) δ = 163.49, 149.85, 148.46, 137.20, 136.83, 132.02, 129.34, 128.96, 128.27, 122.59, 118.25, 107.39, 18.30, 17.39. HRMS (ESI): calcd. for $C_{16}H_{15}N_2S$ $[M + H]^+$ 267.0950, found 267.0950.

E. Reference

1. Lu, C.; Ye, M.; Li, M.; Zhang, Z.; He, Y.; Long, L.; Chen, Z. Transition-metal-switchable divergent synthesis of nitrile-containing pyrazolo[1,5-a]pyridines and indolizines. *Chin. Chem. Lett.*, 2021, 32, 3967-3971.

F. Copies of ^{1}H and ^{13}C NMR spectra

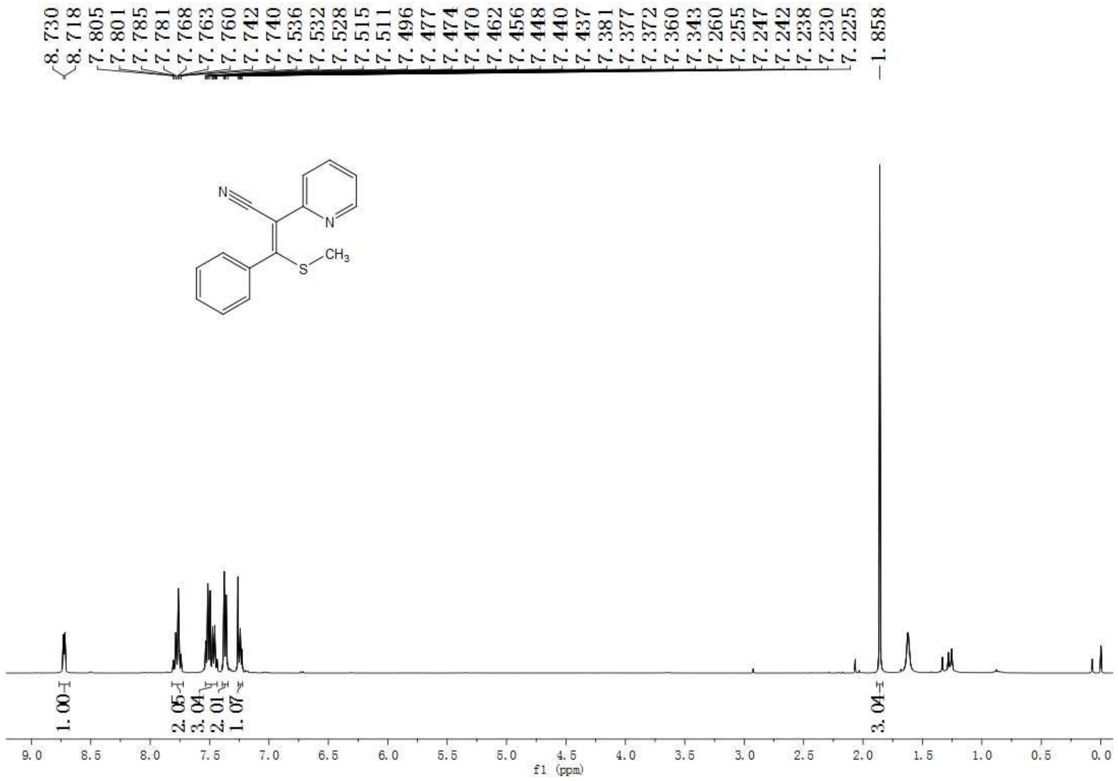

Figure S1. ^{1}H NMR Spectrum of 3a (400 MHz, $CDCl_3$)

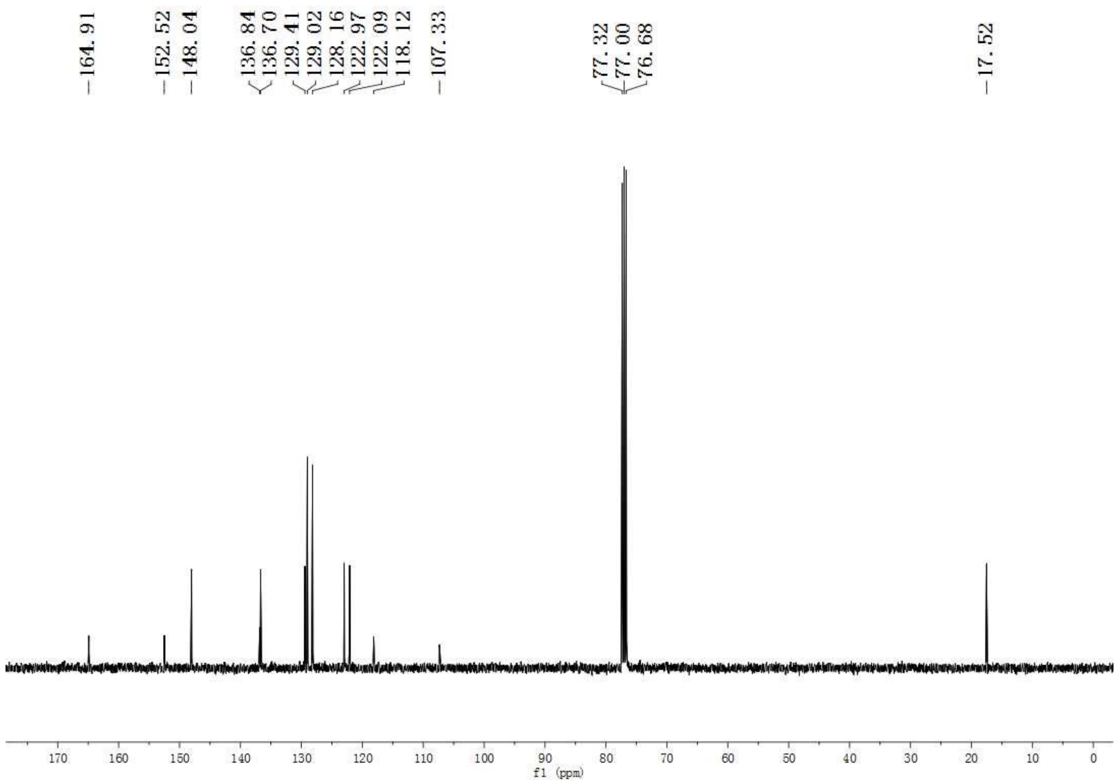

Figure S2. ^{13}C NMR Spectrum of 3a (100 MHz, $CDCl_3$)

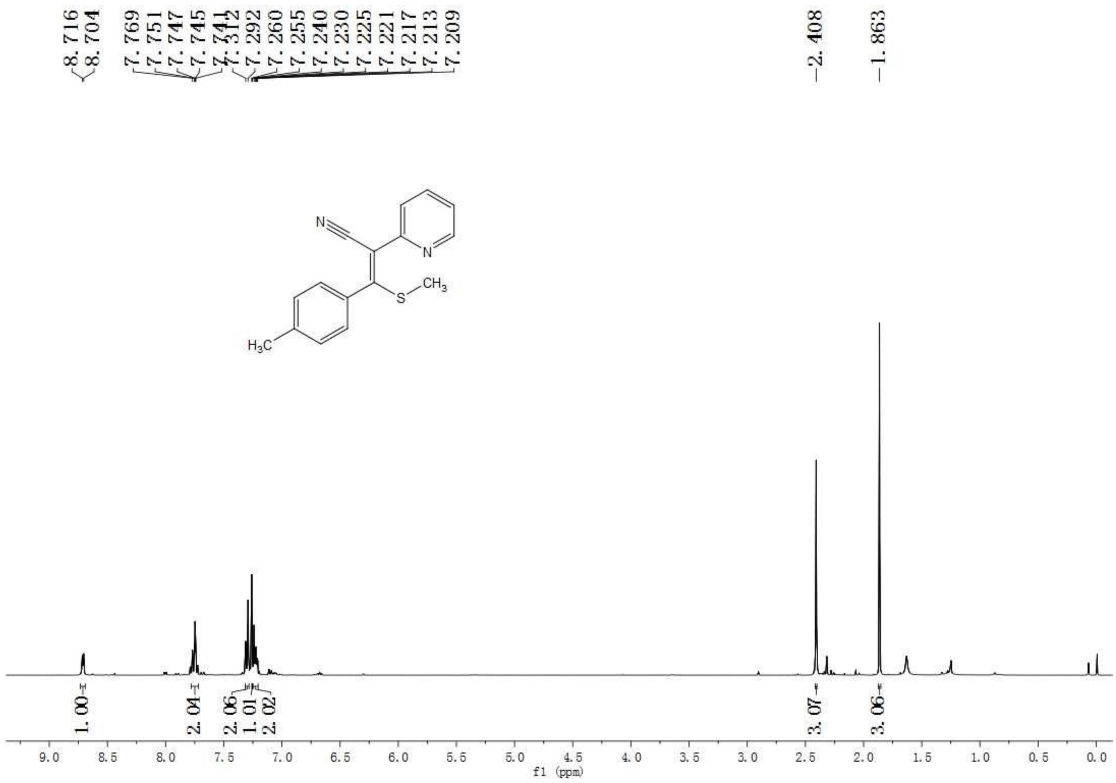

Figure S3. ^{1}H NMR Spectrum of 3b (400 MHz, $CDCl_3$)

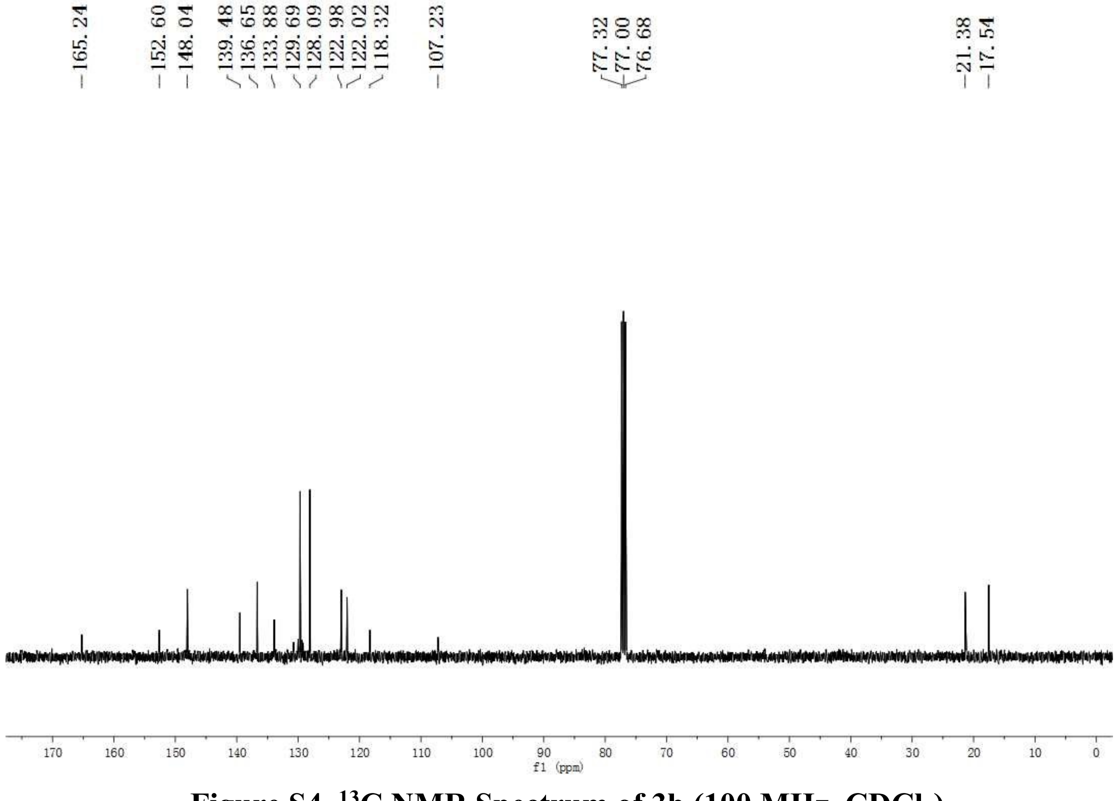

Figure S4. ^{13}C NMR Spectrum of 3b (100 MHz, $CDCl_3$)

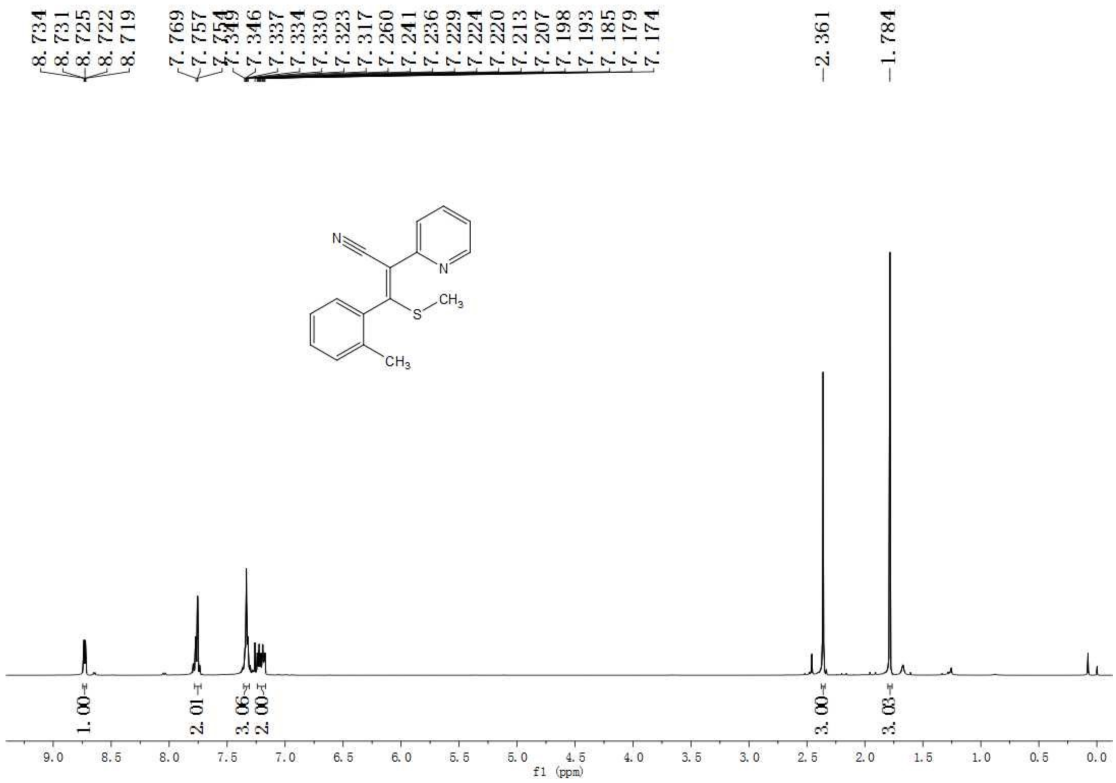

Figure S5. ^{1}H NMR Spectrum of 3c (400 MHz, $CDCl_3$)

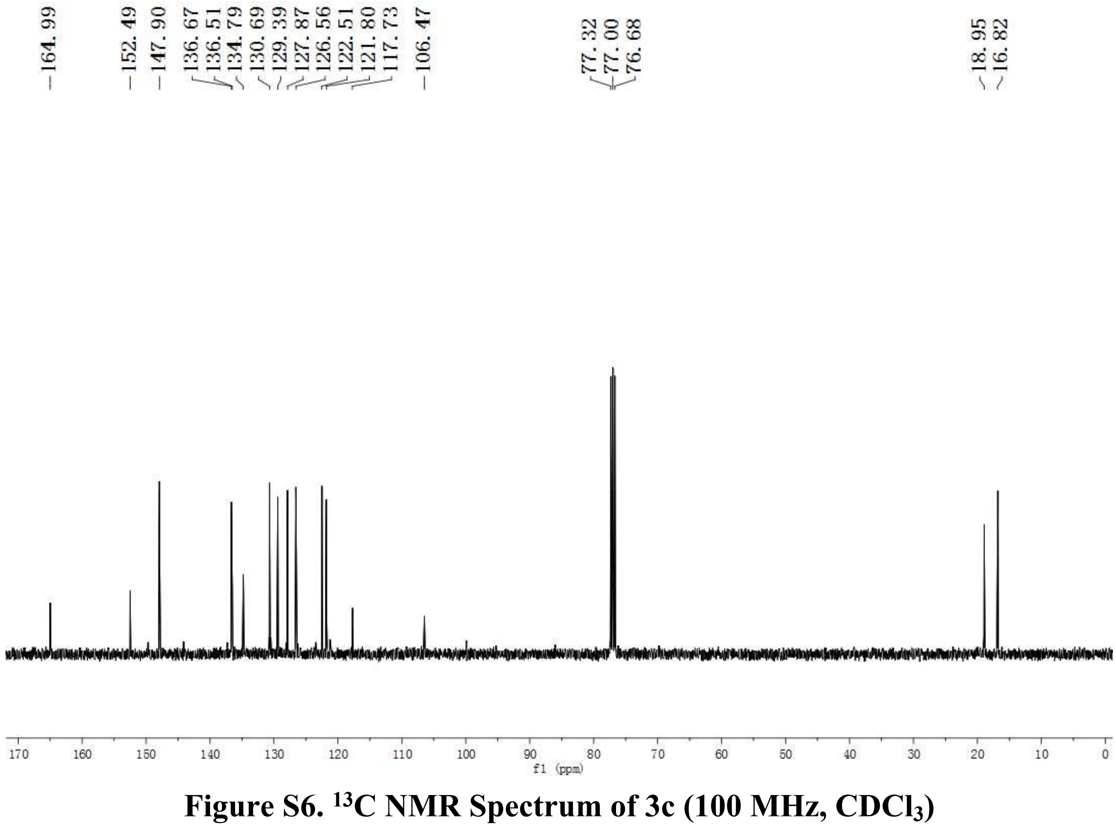

Figure S6. ^{13}C NMR Spectrum of 3c (100 MHz, $CDCl_3$)

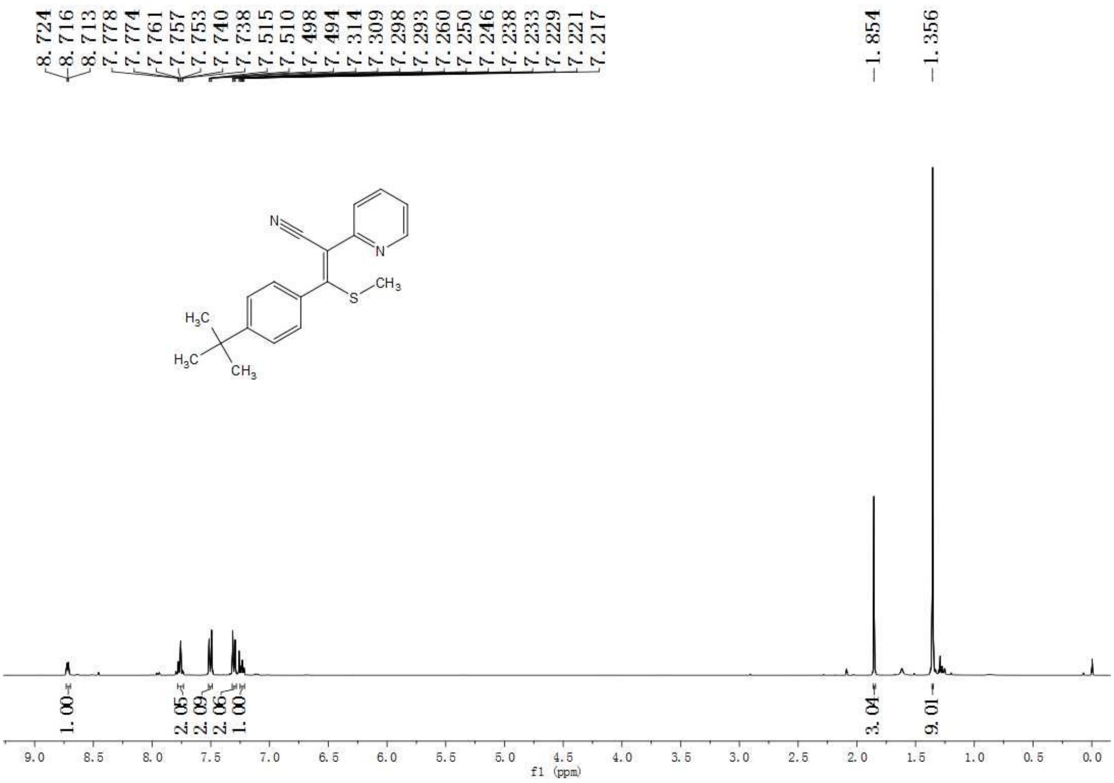

Figure S7. ^{1}H NMR Spectrum of 3d (400 MHz, $CDCl_3$)

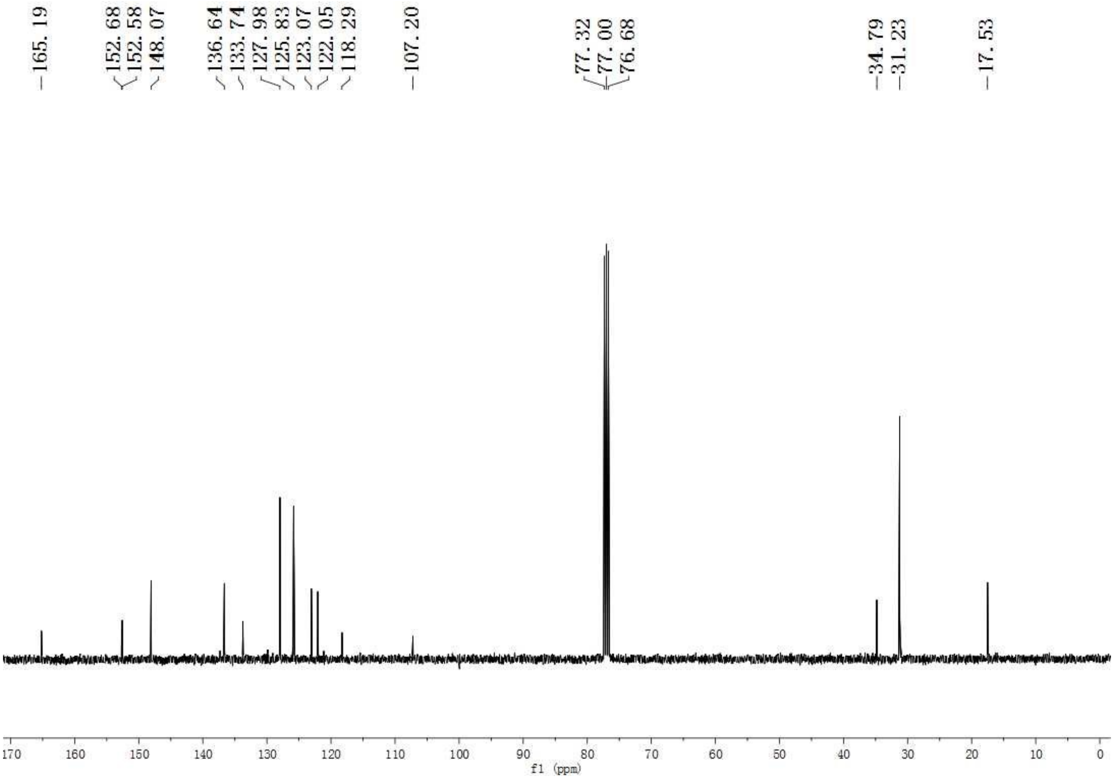

Figure S8. ^{13}C NMR Spectrum of 3d (100 MHz, $CDCl_3$)

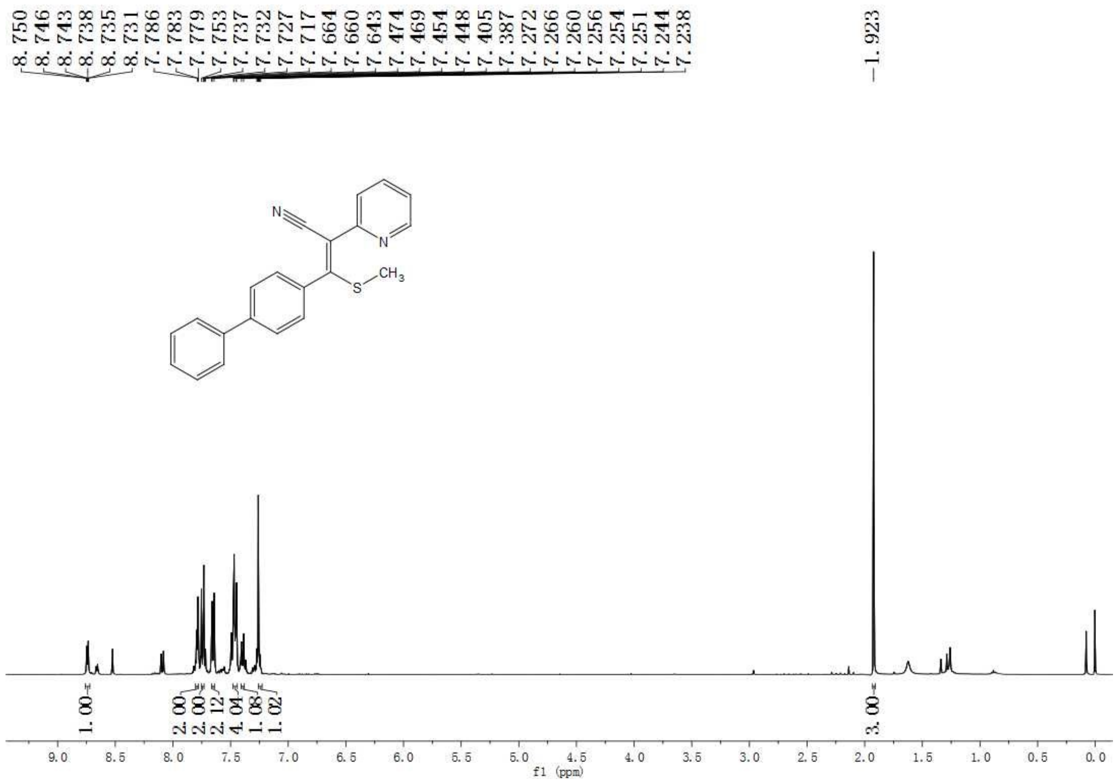

Figure S9. ^{1}H NMR Spectrum of 3e (400 MHz, $CDCl_3$)

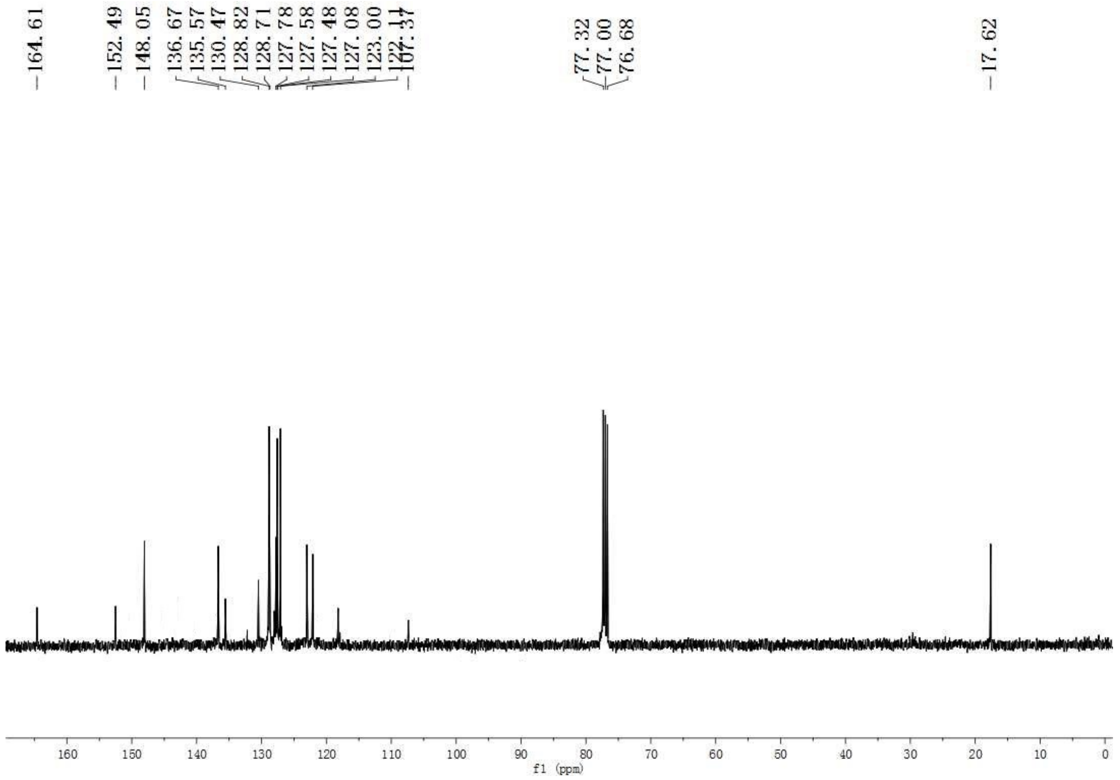

Figure S10. ^{13}C NMR Spectrum of 3e (100 MHz, $CDCl_3$)

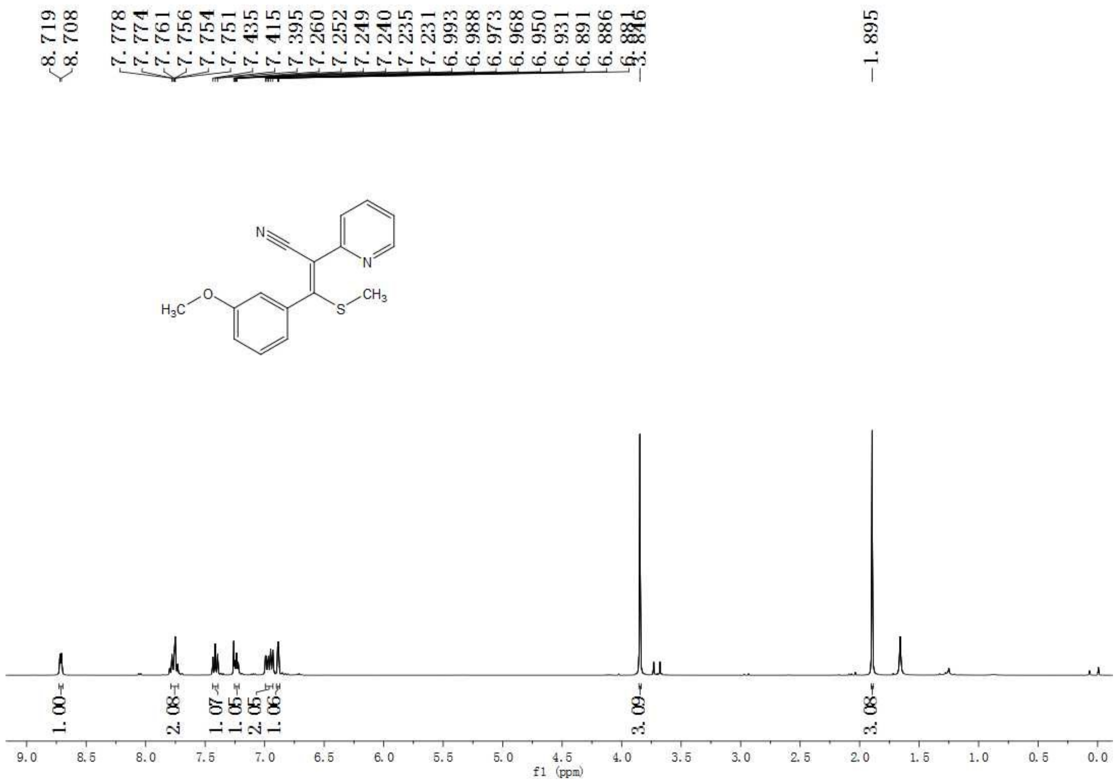

Figure S11. ^{1}H **NMR Spectrum of 3f (400 MHz,** $CDCl_3$**)**

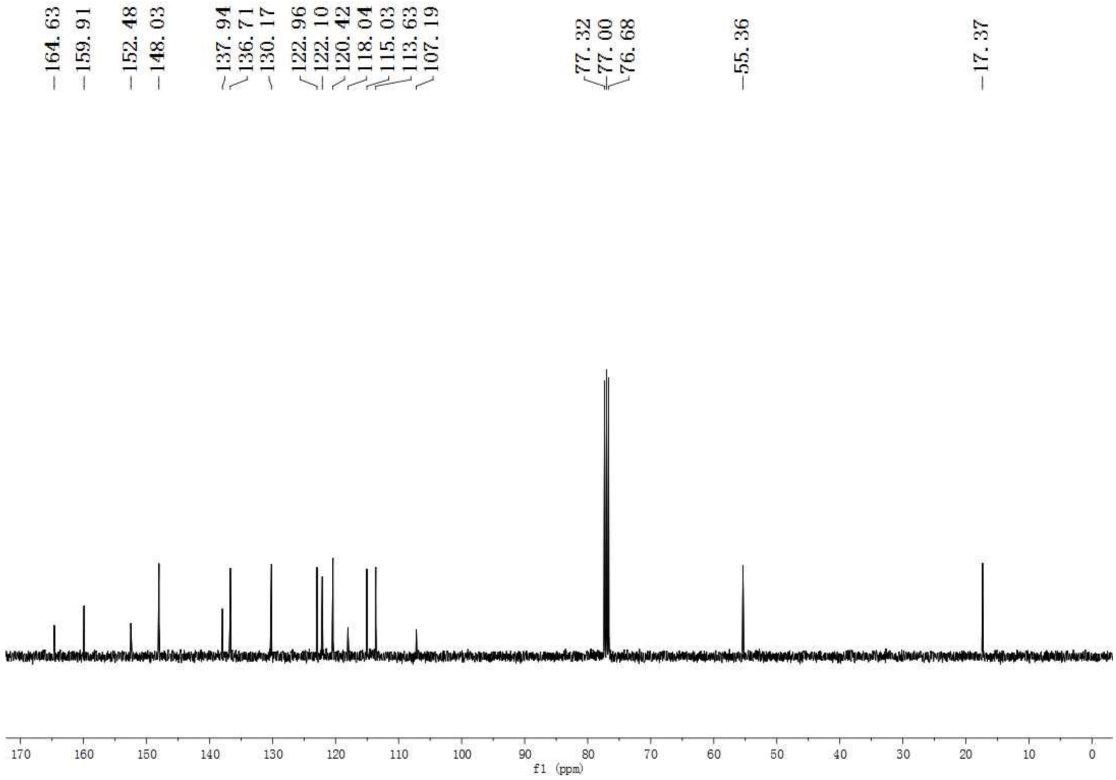

Figure S12. ^{13}C **NMR Spectrum of 3f (100 MHz,** $CDCl_3$**)**

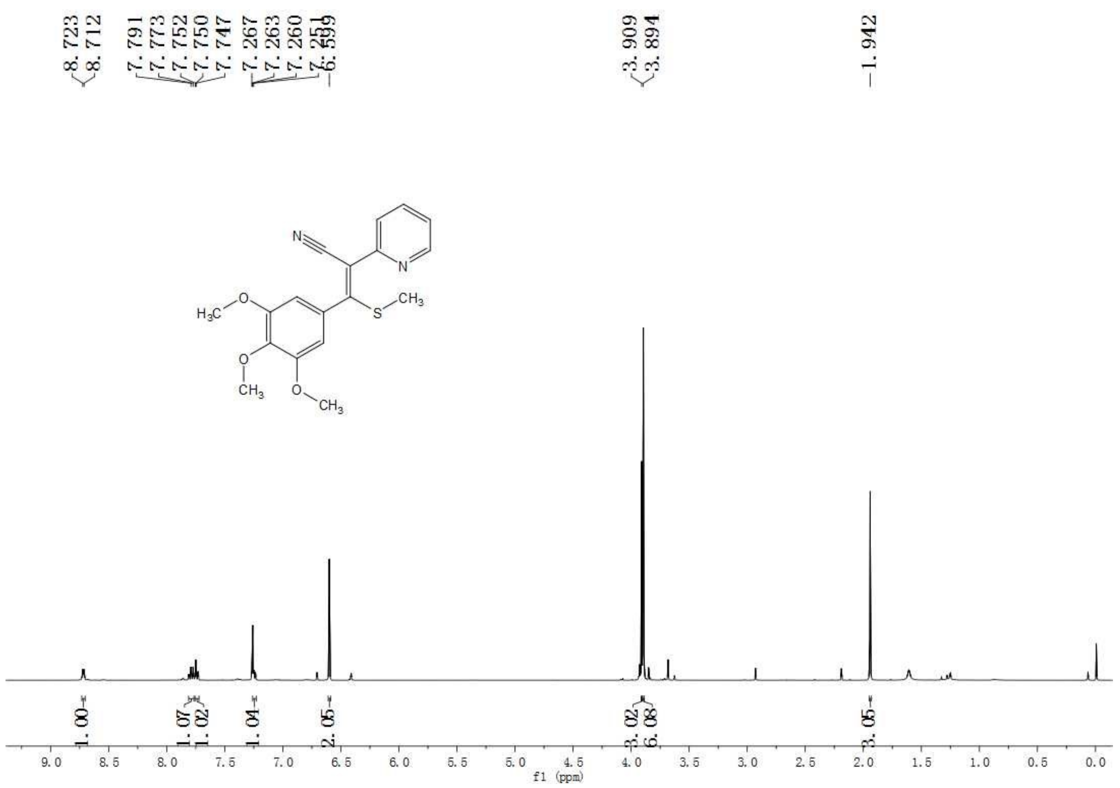

Figure S13. ^{1}H NMR Spectrum of 3g (400 MHz, CDCl$_3$)

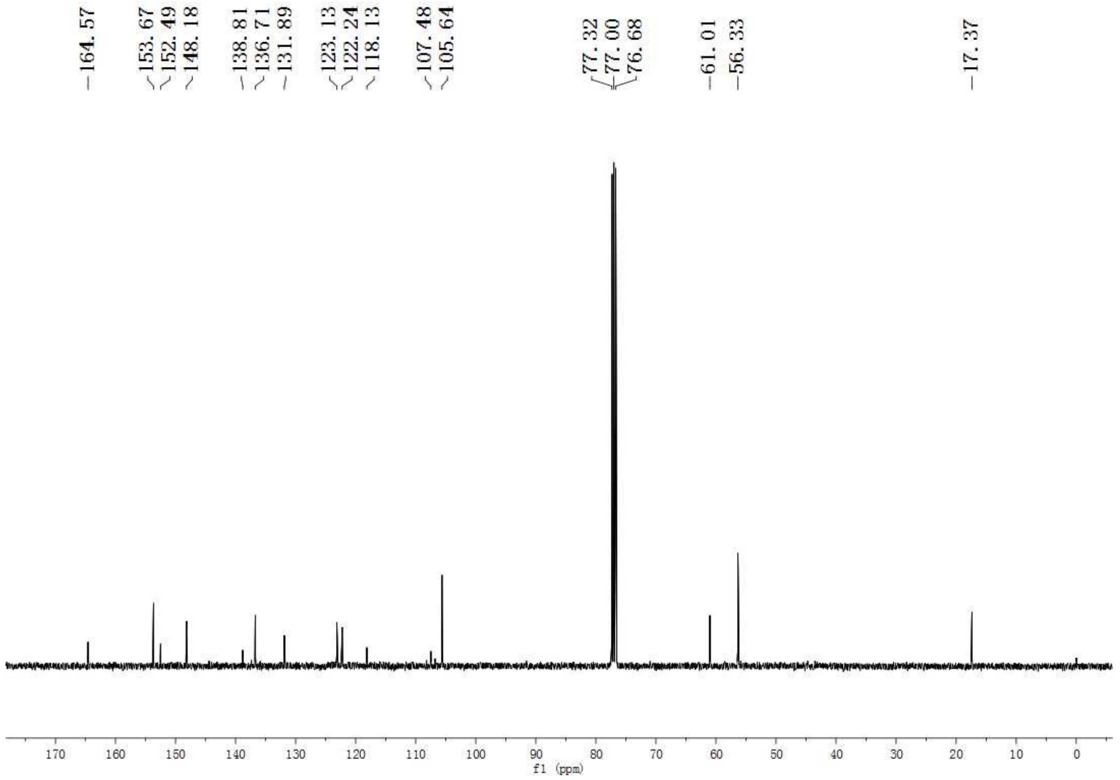

Figure S14. ^{13}C NMR Spectrum of 3g (100 MHz, CDCl$_3$)

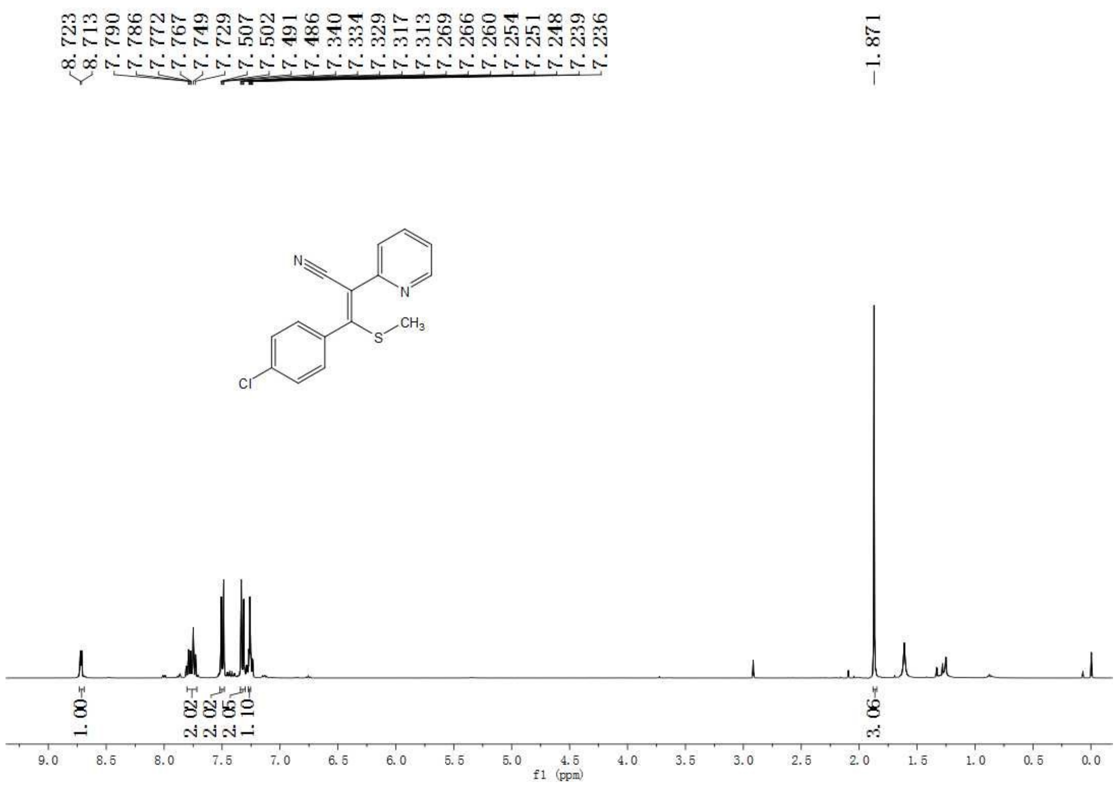

Figure S15. ^{1}H NMR Spectrum of 3h (400 MHz, $CDCl_3$)

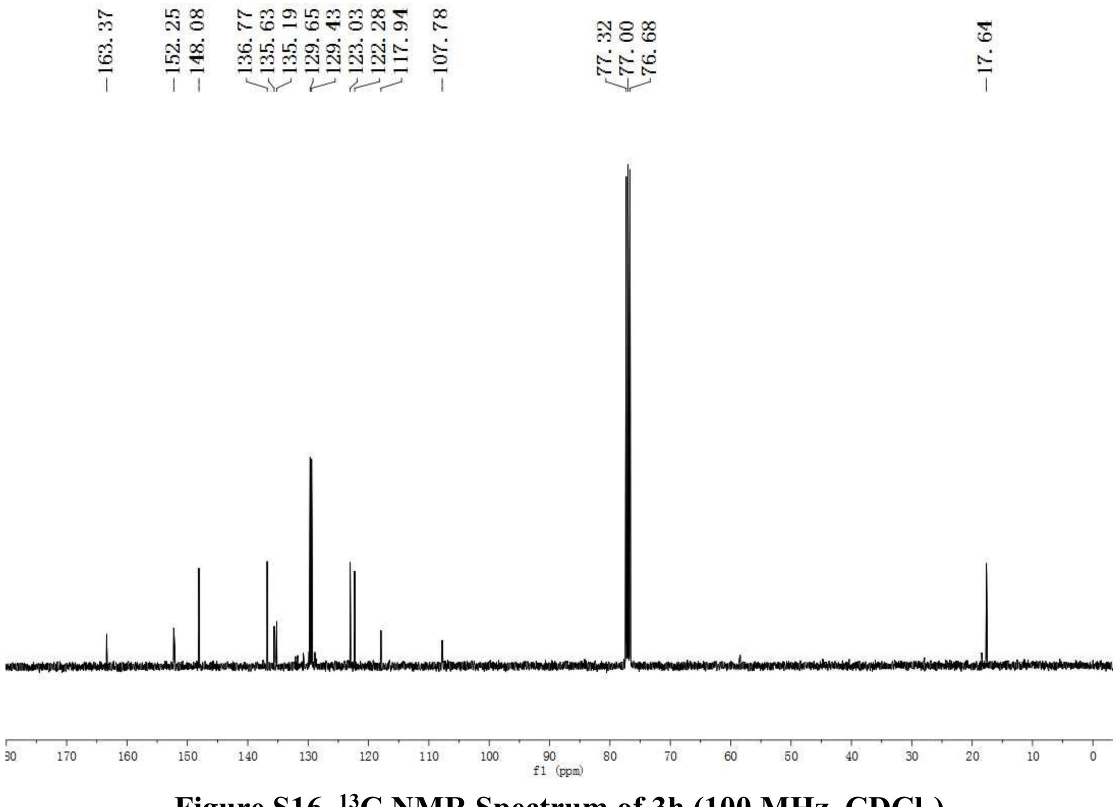

Figure S16. ^{13}C NMR Spectrum of 3h (100 MHz, $CDCl_3$)

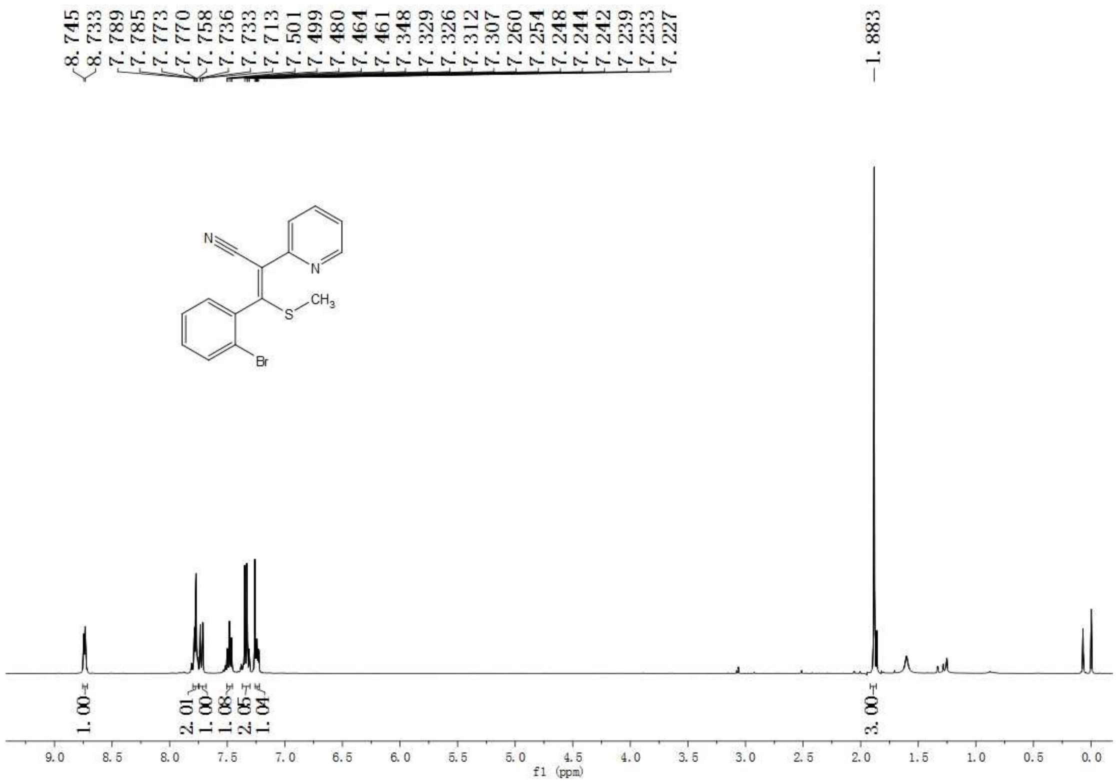

Figure S17. ^{1}H NMR Spectrum of 3i (400 MHz, $CDCl_3$)

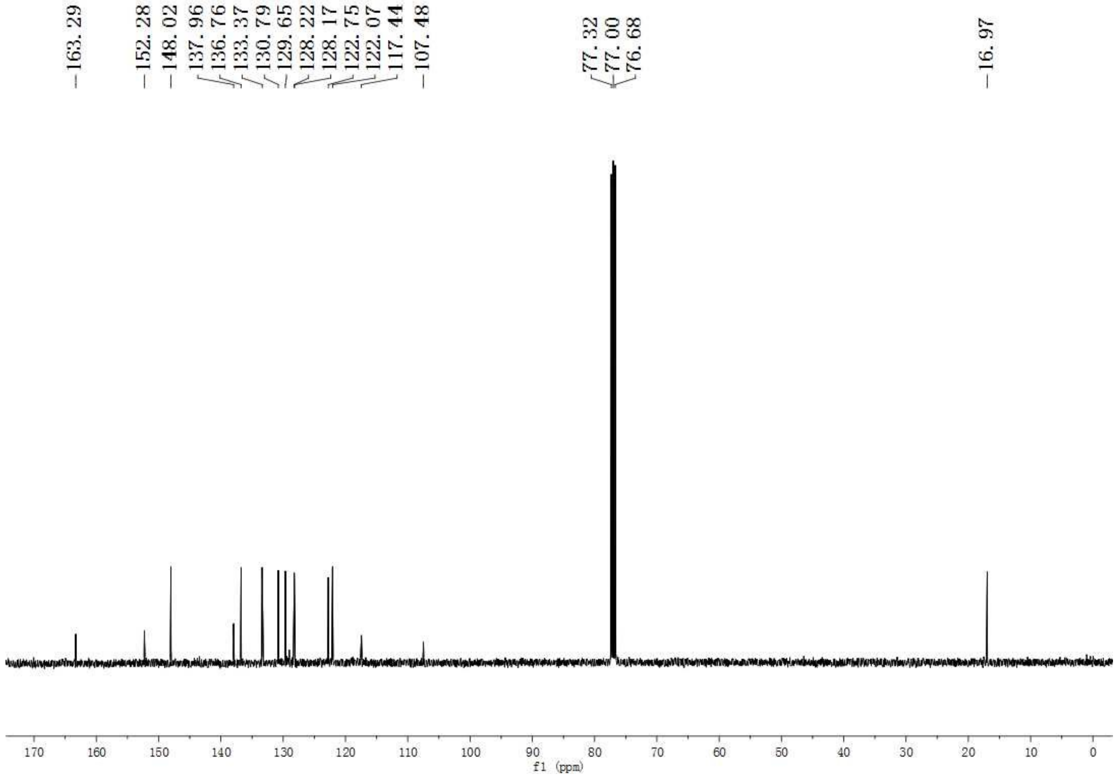

Figure S18. ^{13}C NMR Spectrum of 3i (100 MHz, $CDCl_3$)

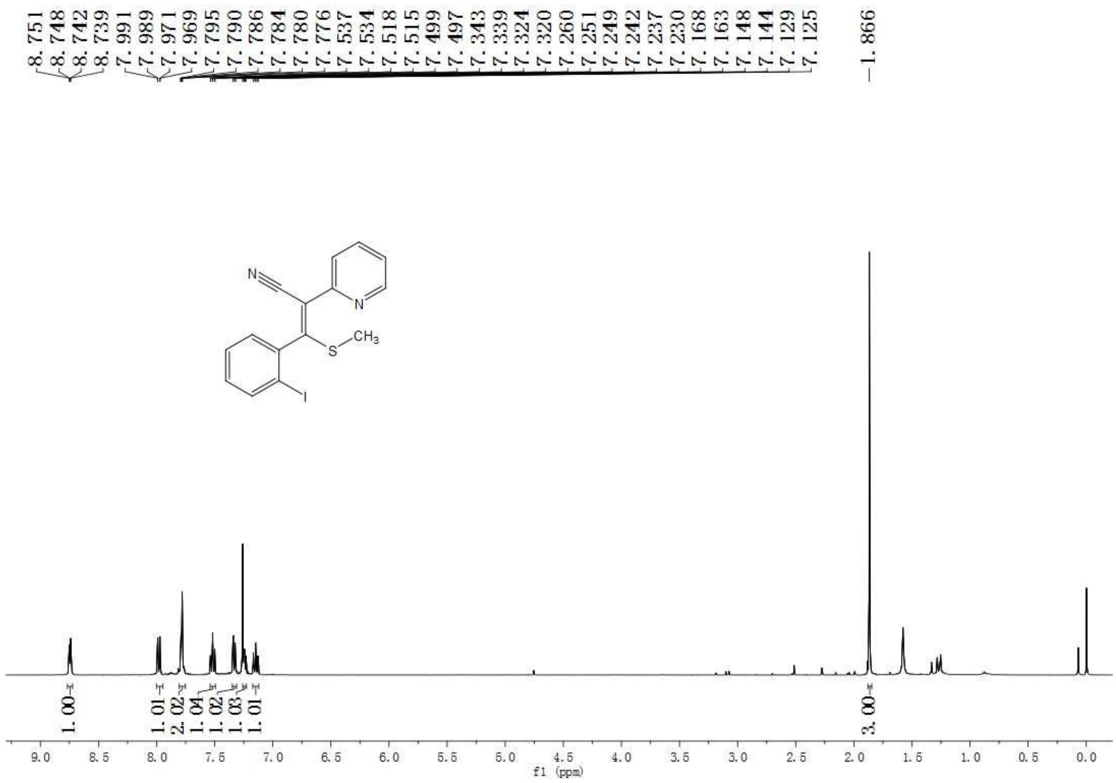

Figure S19. ^{1}H NMR Spectrum of 3j (400 MHz, $CDCl_3$)

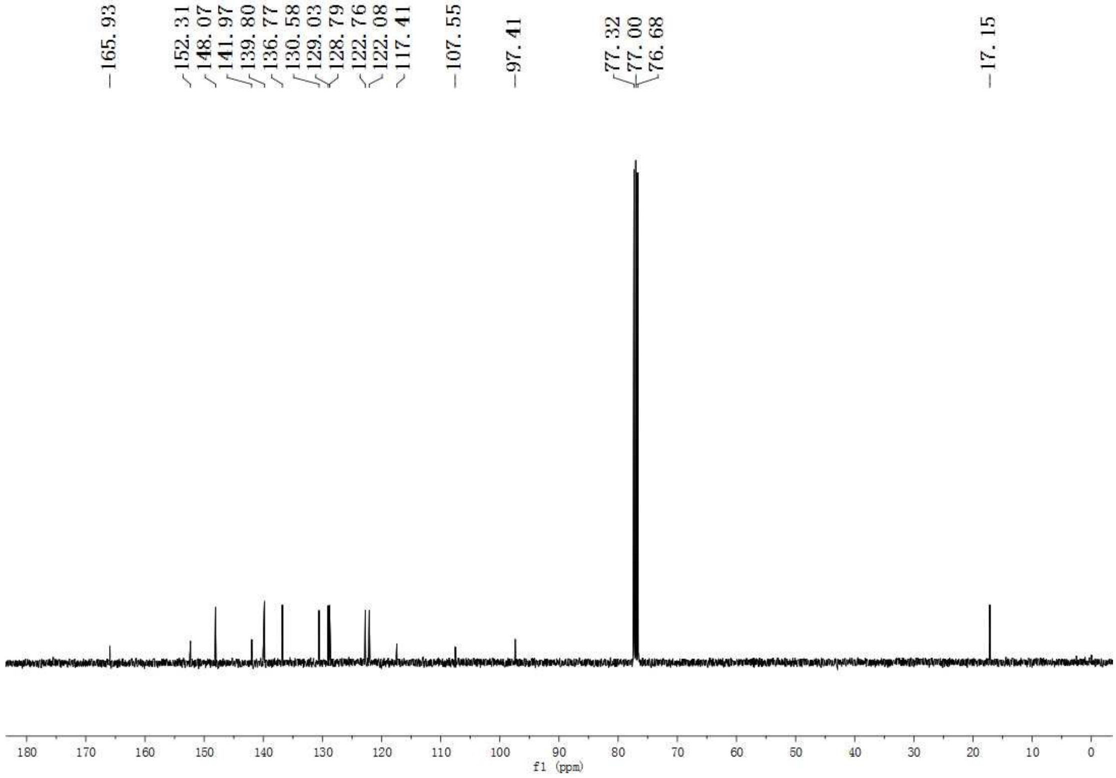

Figure S20. ^{13}C NMR Spectrum of 3j (100 MHz, $CDCl_3$)

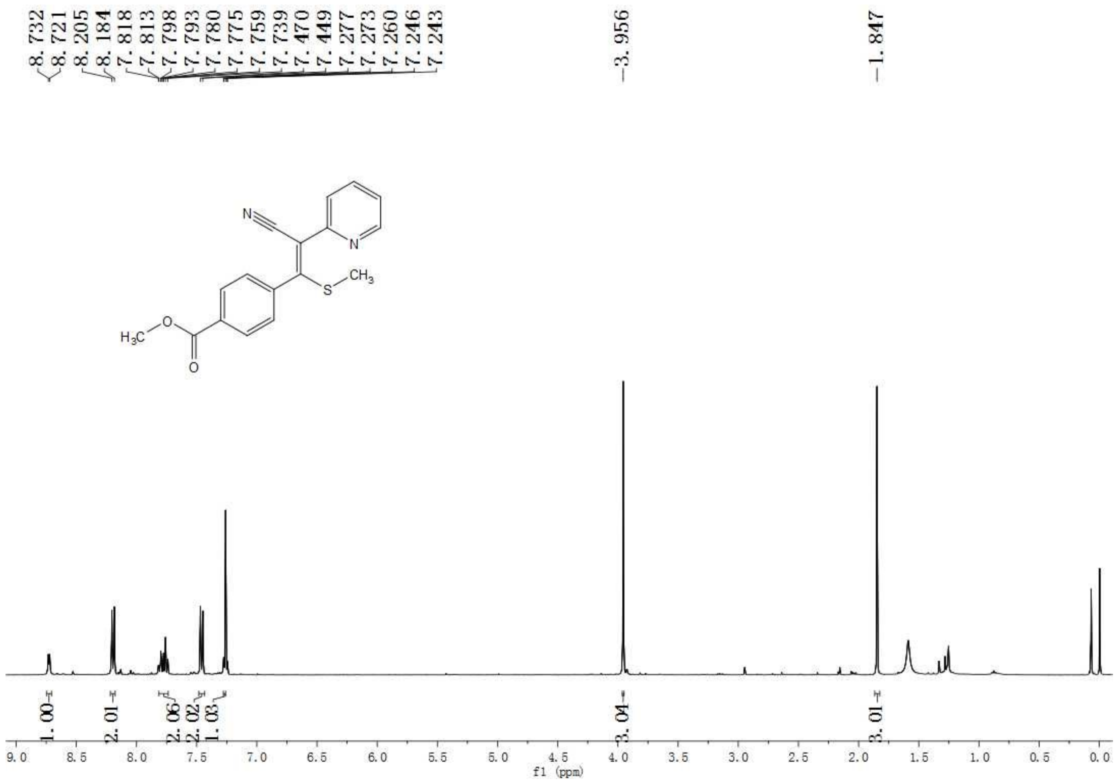

Figure S21. ^{1}H NMR Spectrum of 3k (400 MHz, $CDCl_3$)

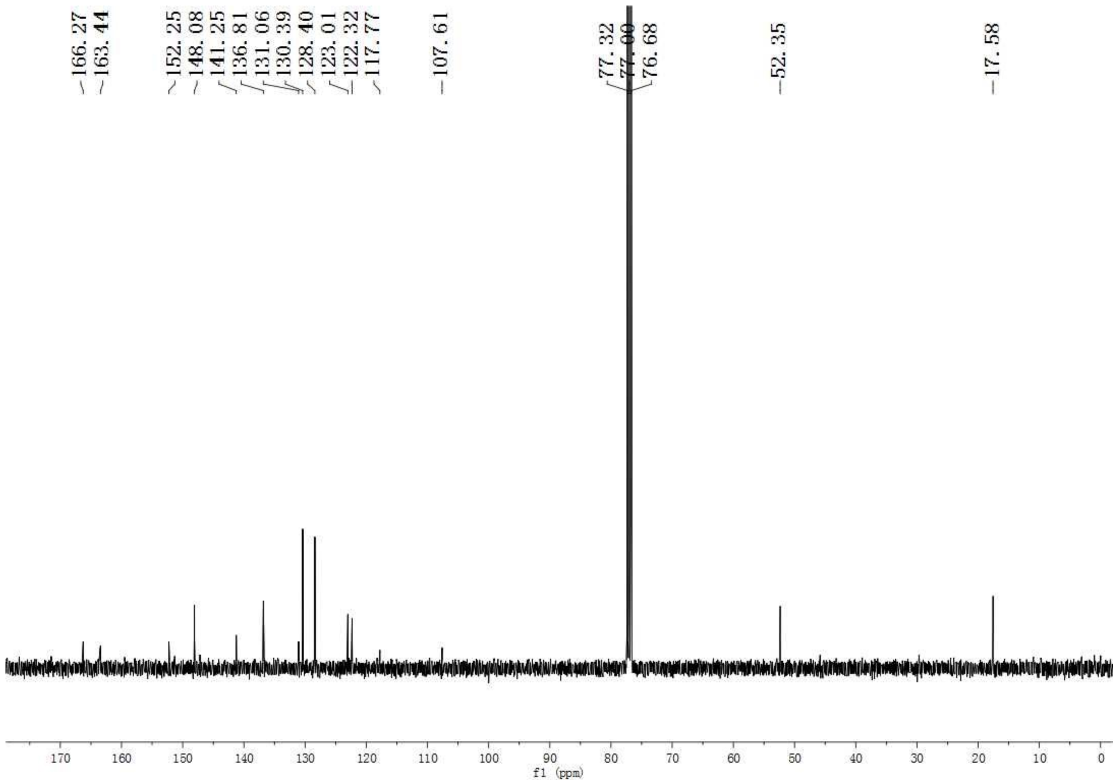

Figure S22. ^{13}C NMR Spectrum of 3k (100 MHz, $CDCl_3$)

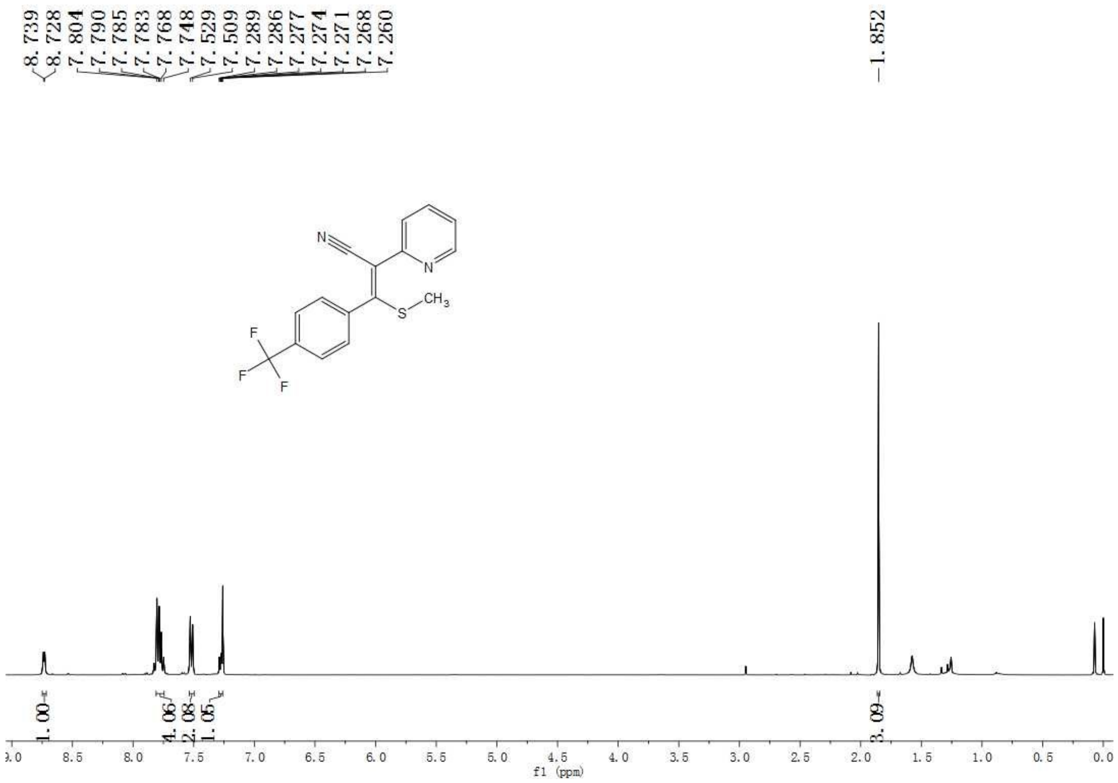

Figure S23. ^{1}H NMR Spectrum of 3l (400 MHz, $CDCl_3$)

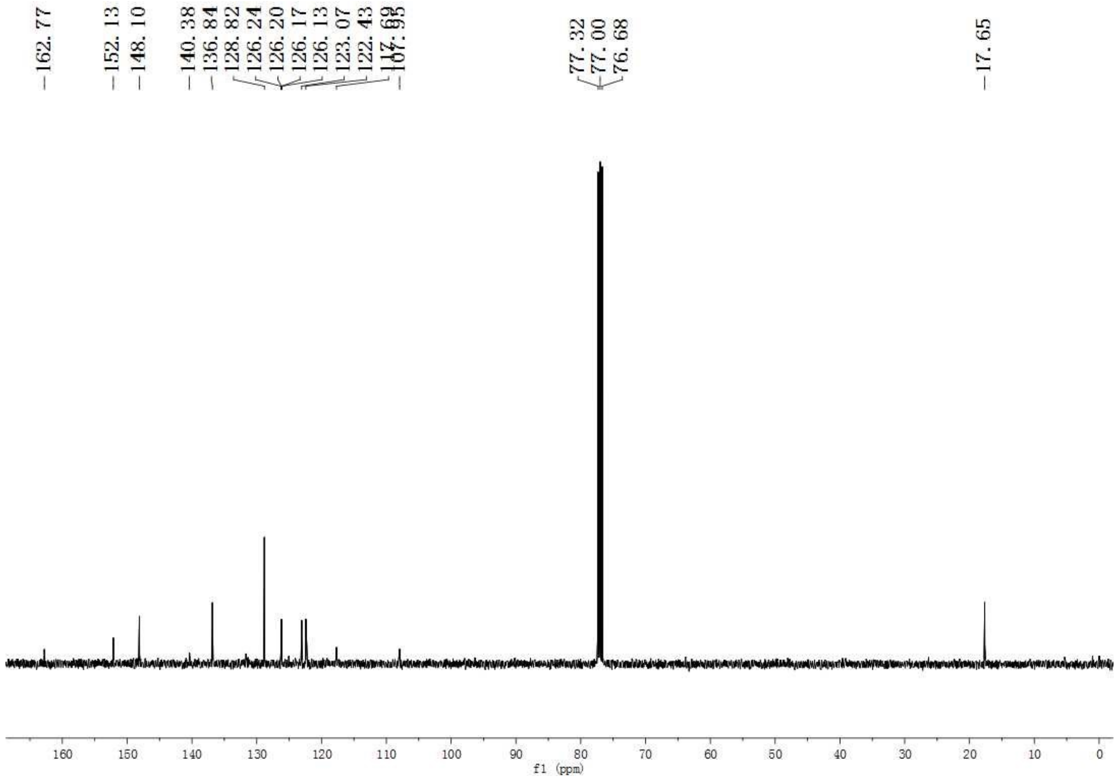

Figure S24. ^{13}C NMR Spectrum of 3l (100 MHz, $CDCl_3$)

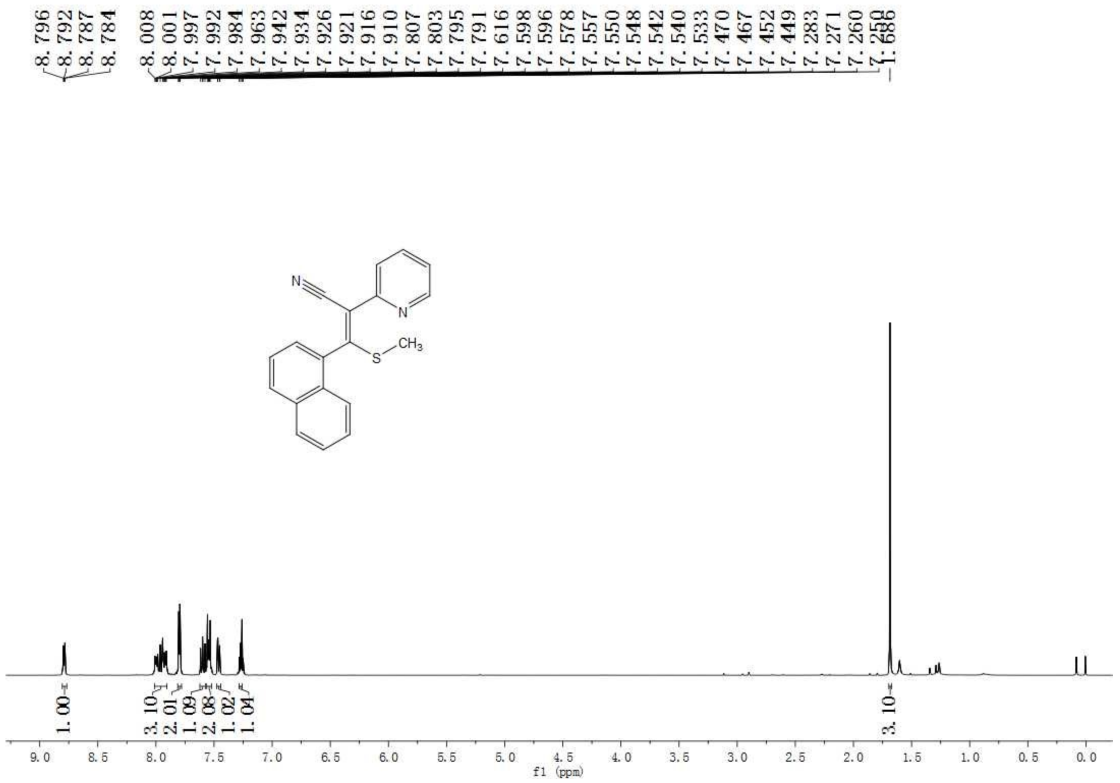

Figure S25. ^{1}H NMR Spectrum of 3m (400 MHz, $CDCl_3$)

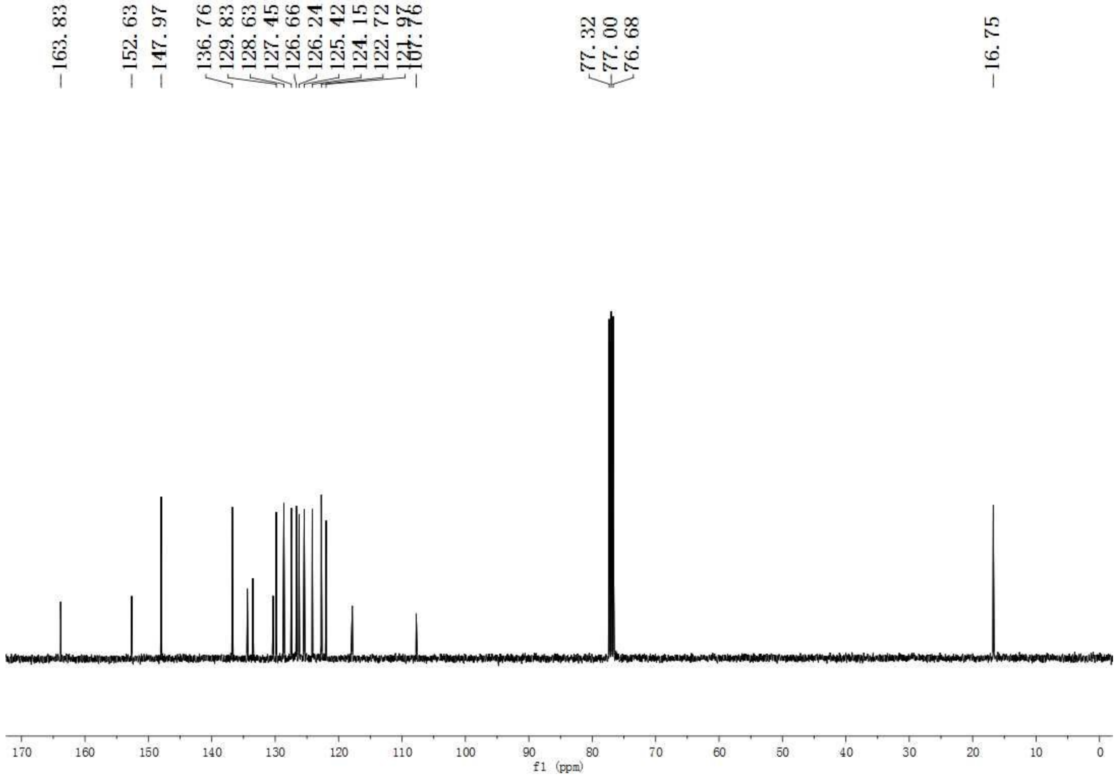

Figure S26. ^{13}C NMR Spectrum of 3m (100 MHz, $CDCl_3$)

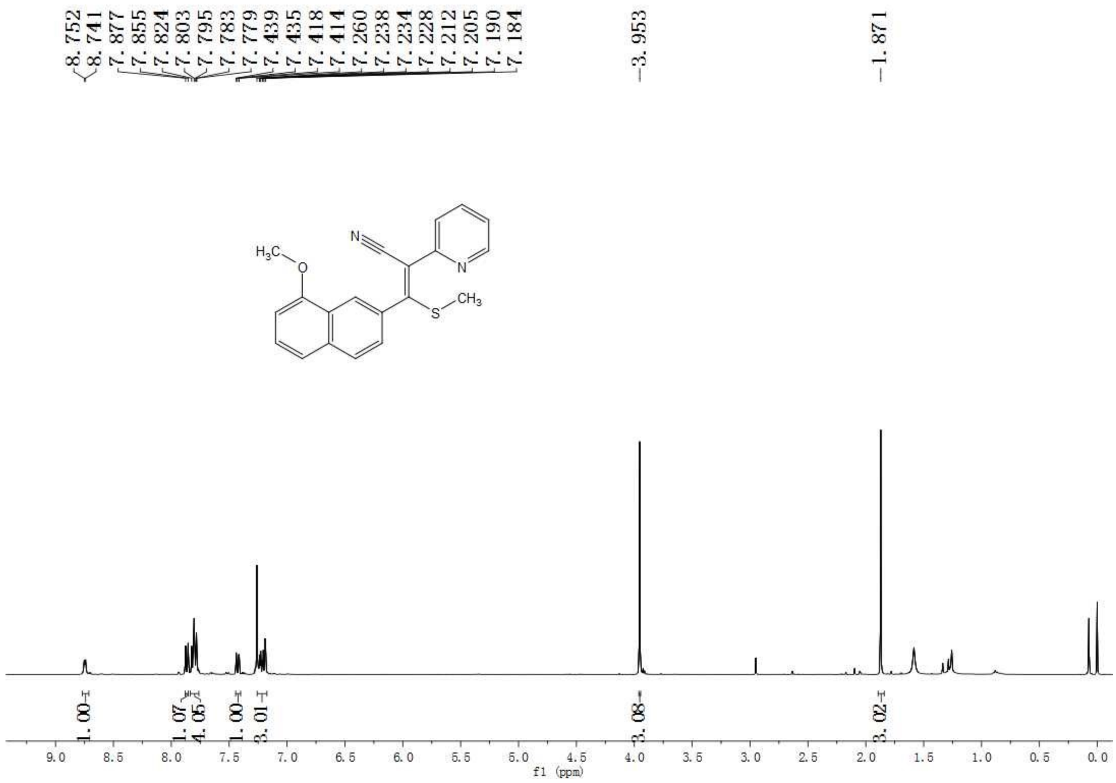

Figure S27. ^{1}H NMR Spectrum of 3n (400 MHz, $CDCl_3$)

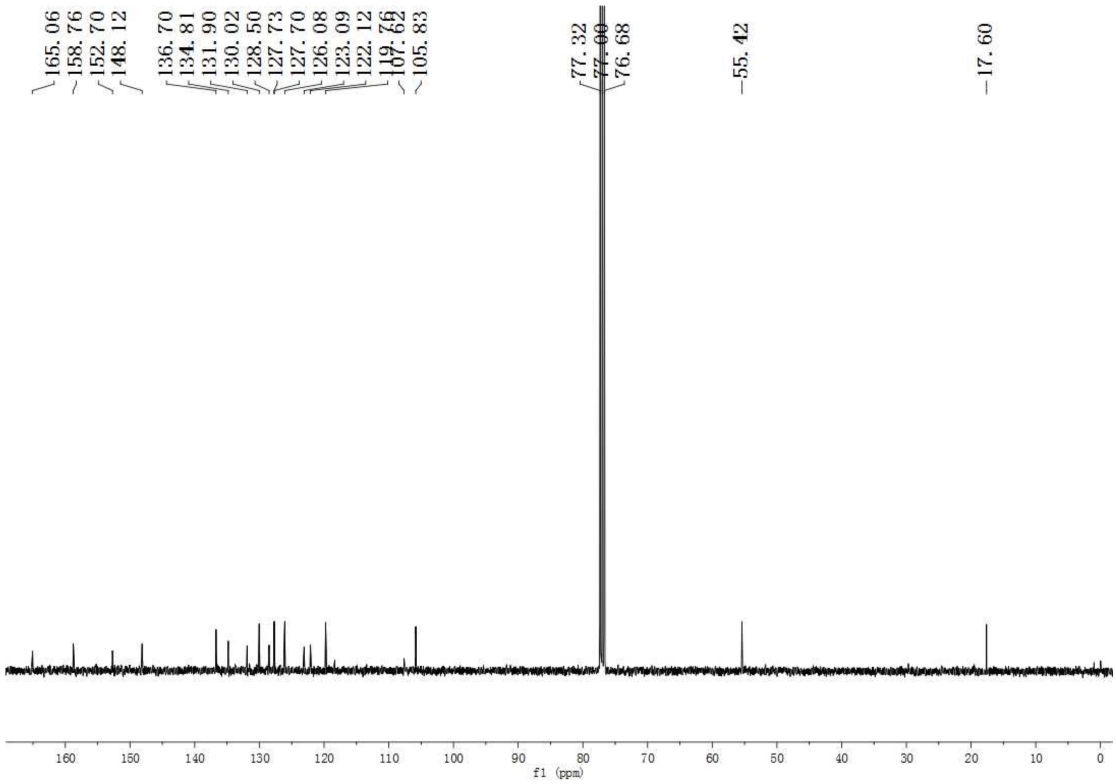

Figure S28. ^{13}C NMR Spectrum of 3n (100 MHz, $CDCl_3$)

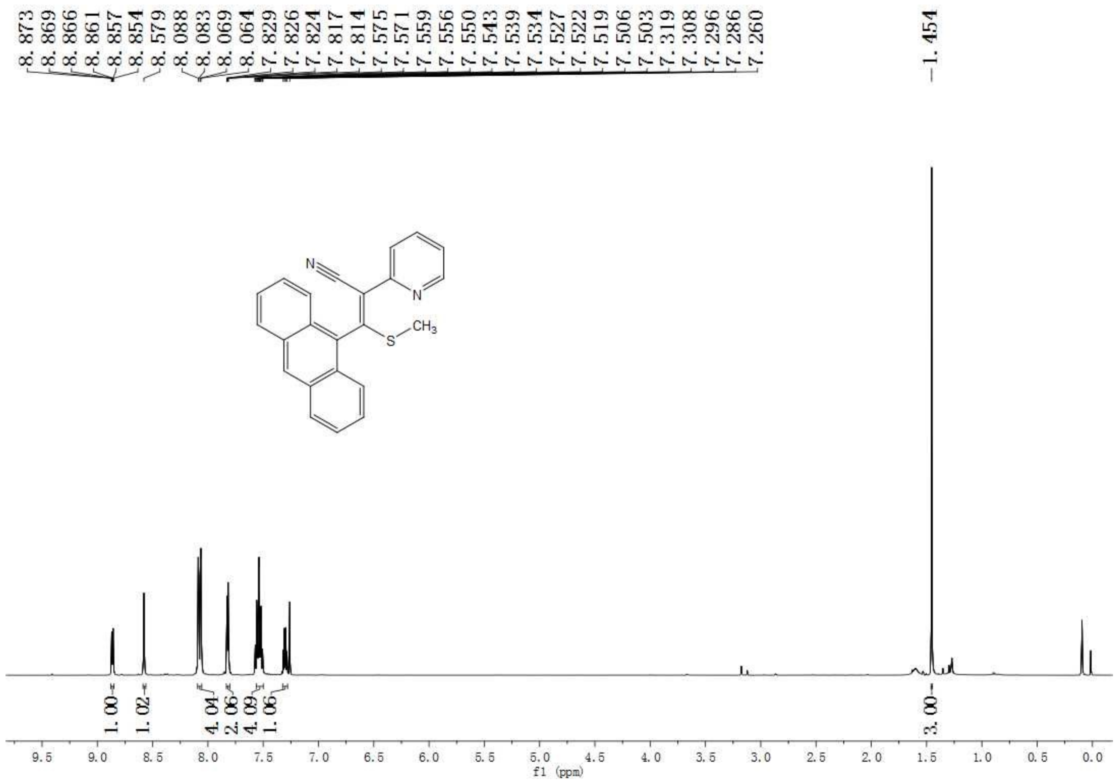

Figure S29. ^{1}H NMR Spectrum of 3o (400 MHz, $CDCl_3$)

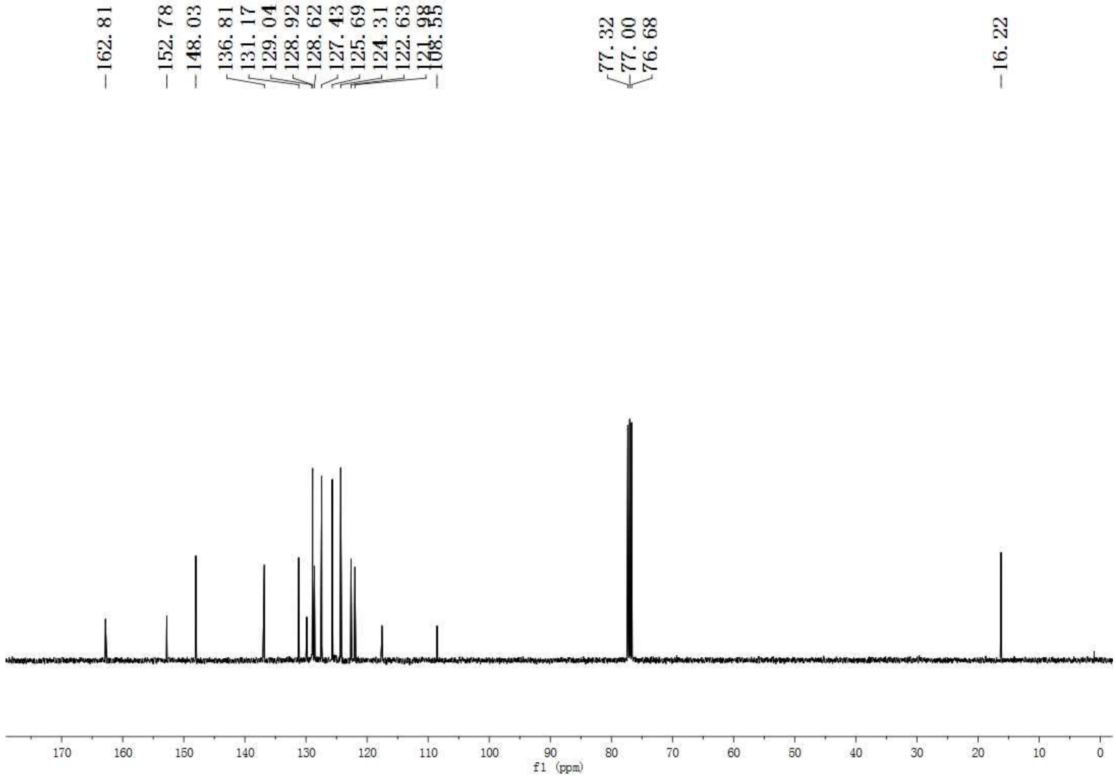

Figure S30. ^{13}C NMR Spectrum of 3o (100 MHz, $CDCl_3$)

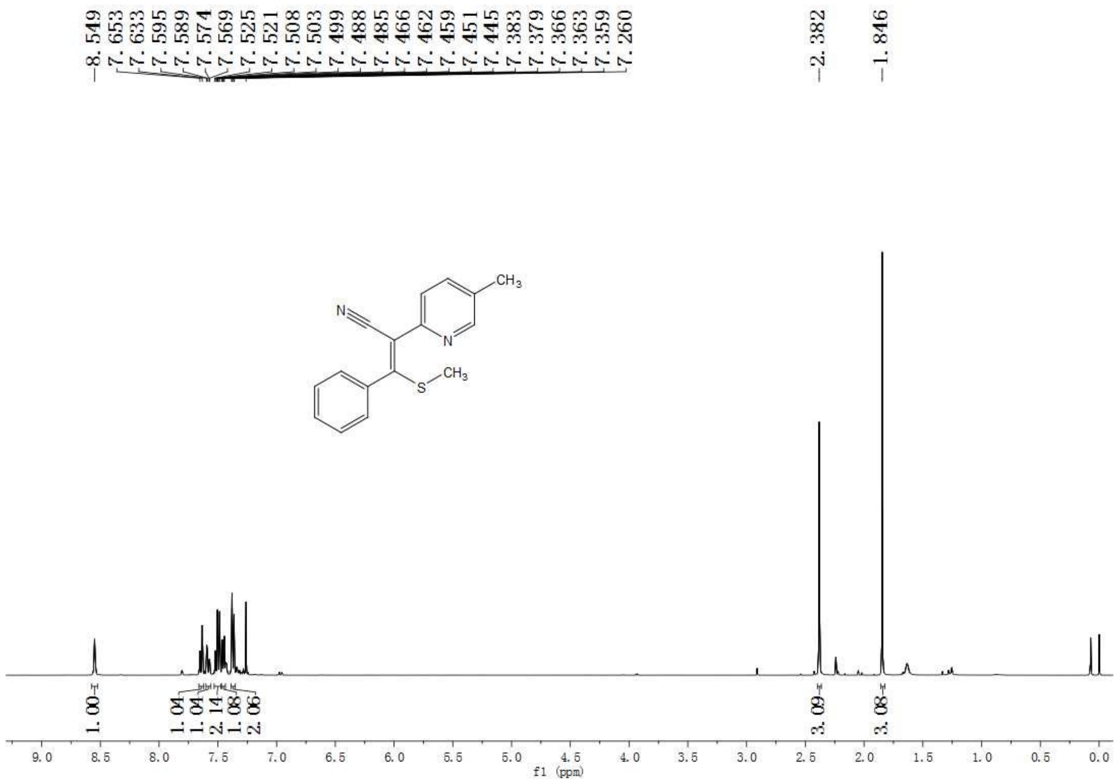

Figure S31. ^{1}H NMR Spectrum of 3p (400 MHz, $CDCl_3$)

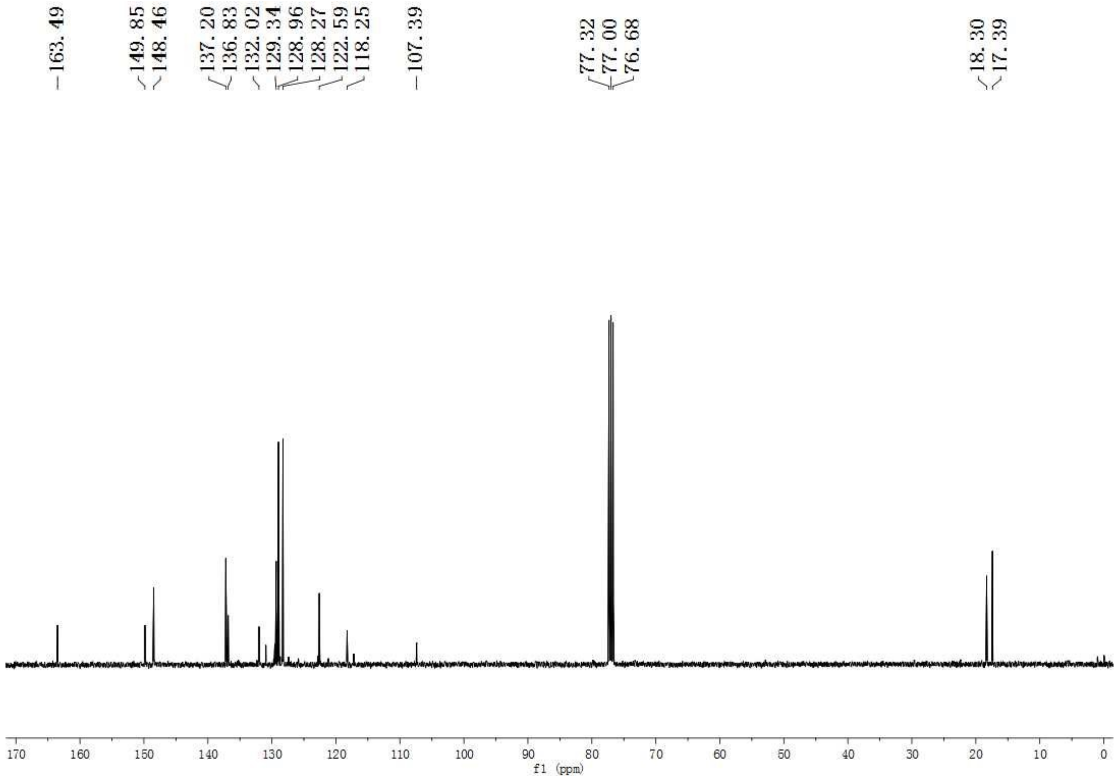

Figure S32. ^{13}C NMR Spectrum of 3p (100 MHz, $CDCl_3$)
